# Further characterization of adult sheep ovarian stem cells and their involvement in neo-oogenesis and follicle assembly

**DOI:** 10.1186/s13048-017-0377-5

**Published:** 2018-01-05

**Authors:** Hiren Patel, Deepa Bhartiya, Seema Parte

**Affiliations:** Stem Cell Biology Department, ICMR-National Institute for Research in Reproductive Health, Jehangir Merwanji Street, Parel, Mumbai, 400 012 India

**Keywords:** Ovary, Stem cells, FSH, Primordial follicle, VSELs, OCT-4, FSHR

## Abstract

**Background:**

Stem cells in the ovary comprise of two distinct populations including very small embryonic-like stem cells (VSELs) and slightly bigger progenitors termed ovarian stem cells (OSCs). They are lodged in ovary surface epithelium (OSE) and are expected to undergo neo-oogenesis and primordial follicle (PF) assembly in adult ovaries. The ovarian stem cells express follicle stimulating hormone (FSH) receptors and are directly activated by FSH resulting in formation of germ cell nests (GCN) in vitro. Present study was undertaken to further characterize adult sheep OSCs and to understand their role during neo-oogenesis and PF assembly.

**Methods:**

Stem cells were collected by gently scraping the OSE cells and were characterized by H&E staining, immuno-localization, immuno-phenotyping and RT-PCR studies. Expression of FSH receptors and markers specific for stem cells (OCT-4, SSEA-4) and proliferation (PCNA) were studied on stem/progenitor cells in OSE culture and on adult sheep ovarian cortical tissue sections. Effect of FSH on stem cells was also studied in vitro. Asymmetric cell division (ACD) was monitored by studying expression of OCT-4 and NUMB.

**Results:**

Additional evidence was generated on the presence of two populations of stem cells in the OSE including VSELs and OSCs. FSHR expression was observed on both VSELs and OSCs by immuno-localization and immuno-phenotyping studies. FSH treatment in vitro stimulated VSELs that underwent ACD to self-renew and give rise to OSCs which divided rapidly by symmetric cell divisions (SCD) and clonal expansion with incomplete cytokinesis to form GCN. ACD was further confirmed by differential expression of OCT-4 in VSELs and NUMB in the OSCs. Immuno-histochemical expression of OCT-4, PCNA and FSHR was noted on stem cells located in the OSE in sheep ovarian sections. GCN and cohort of PF were observed in the ovarian cortex and provided evidence in support of neo-oogenesis from the stem cells.

**Conclusion:**

Results of present study provide further evidence in support of two stem cells populations in adult sheep ovary. Both VSELs, OSCs and GCN express FSH receptors and FSH possibly regulates their function to undergo neo-oogenesis and primordial follicle assembly.

**Electronic supplementary material:**

The online version of this article (10.1186/s13048-017-0377-5) contains supplementary material, which is available to authorized users.

## Background

General consensus exists among reproductive biologists that a mammalian ovary has finite number of follicles at birth that get depleted over time and a sudden depletion of follicles with age leads to menopause [1]. Several groups have reported ovarian stem cells (OSCs) in adult mammalian ovary that are expected to undergo neo-oogenesis and primordial follicle assembly just like spermatogenesis in testis and menopause occurs probably due to compromised somatic niche with age that becomes unable to support normal differentiation of stem cells and primordial follicle assembly [2, 3]. However, a consensus on the very existence of OSCs in adult ovaries [4] and evidence in support of their in vivo role during neo-oogenesis and follicle assembly is yet to emerge. Our group recently made an attempt to resolve existing controversies in the field of ovarian stem cells [5].

Presence of OSCs and neo-oogenesis was first reported by Tilly’s group in 2004 who demonstrated the presence of 5–8 μm OSCs in ovary surface epithelium (OSE) and the work done on these stem cells was compiled [6, 7]. Virant-Klun’s group has reported the presence of smaller 3–5 μm SSEA-4 positive stem cells in human OSE expressing several pluripotent and primordial germ cells (PGCs) specific markers and their ability to spontaneously differentiate into oocytes-like structures in vitro [8, 9]. Our group reported the presence of two distinct populations of stem cells in OSE comprising smaller 3–5 μm very small embryonic-like stem cells (VSELs) expressing nuclear OCT-4 and other pluripotent and PGCs specific markers and another population of slightly bigger 5–8 μm progenitors which were termed ovarian germ stem cells (OGSCs) and are similar to OSCs reported by Tilly’s group (Additional file 1: Figure S1) and will be henceforth termed OSCs in the manuscript. The OSCs are reported to express cytoplasmic OCT-4 in humans, sheep, marmoset and rabbit ovaries [10, 11] and also in mouse ovaries [12]. Presence of two distinct populations of stem cells in the OSE was recently discussed [5, 13] and the methods to isolate and characterize both populations of stem cells including primitive VSELs and progenitor OSCs have been published [14]. Similar VSELs were also reported in human [15] and mouse [16–18] testes which exist as a sub-group amongst the well-studied spermatogonial stem cells (SSCs). Thus a similar population of VSELs exists along with OSCs in ovary and SSCs in the testis. VSELs are the most primitive, relatively quiescent, epiblast derived pluripotent stem cells equivalent to primordial germ cells (PGCs) that survive in adult gonads in few numbers and were recently reviewed [19, 20]. OSCs/SSCs are tissue specific progenitors that are bigger in size and have a distinct gene expression pattern compared to pluripotent VSELs. Data is now emerging suggesting that tissue specific stem cells like SSCs in testis [17] and hematopoietic stem cells (HSCs) in the bone marrow [21] arise by asymmetric cell division (ACD) of VSELs and in turn undergo symmetric cell divisions (SCD) and clonal expansion (sphere formation) before differentiating into tissue specific cell types including sperm in testis and blood cells in the bone marrow. How do these stem cells function in vivo in adult mammalian ovaries and what intrinsic factors activate and regulate their biology? These are questions that need to be answered.

Hypothalamus and pituitary gonadal axis plays an important role in both male and female reproductive biology. Pituitary gonadotropin, follicle stimulating hormone (FSH) is central to mammalian reproduction and is known for its pleiotropic action not only in gonads but also in extra gonadal tissues [22, 23]. FSH acts through its receptors (FSHR) that are reported to have splice variant isoforms [24]. We have earlier reported that FSH exerts a direct action on the stem cells both in vivo in mice ovaries [12] and testis [17] as well as in vitro on sheep [11], mouse [12] marmoset and human [10] ovarian and mouse testicular [17] cells. FSH stimulates VSELs to undergo ACD to give rise to SSCs which proliferate and undergo clonal expansion as chains in testis and give rise to OSCs that undergo germ cell nest formation in the ovary. FSHR expression has also been reported on stem/progenitor cells in both ovary and testis [11, 17]. These results were arrived at using 3 different FSHR antibodies, by peptide blocking to confirm specificity of FSHR expression on stem cells and also by qRT-PCR and in situ hybridization at mRNA level [15].

It is intriguing to note that VSELs and HSCs in the hematopoietic system also express FSHR in mouse bone marrow [21–25] as well as in human cord blood [26, 27]. Shaikh et al. [28] reported FSHR on bone marrow stem cells and that FSH treatment to chemoablated (5-flourouracil, 150 mg/Kg) mice augmented the process of bone marrow re-colonization by 72 h. Zbucka-Kretowska et al. [27] reported that VSELs and HSCs (and not endothelial progenitors) are mobilized into the peripheral blood of female patients undergoing FSH therapy for stimulating ovaries in an IVF clinic. These findings suggest that a common pool of VSELs exists in various adult organs with a developmental link to PGCs and this explains FSHR expression in non-gonadal tissues as well.

Difference of opinion exists in the literature on the presence of OSCs due to the use of DDX as a marker to sort OSCs for various studies [29]. However, these controversies surrounding the presence of OSC are purely technology related and stem cells do exist in adult ovaries. Generally, the ovarian tissue is subjected to enzymatic digestion and then centrifuged to obtain a cell pellet (containing stem cells) for various experiments. The OSCs lodged in the OSE get diluted in overall population of cells obtained after digestion and VSELs (which exist in very few numbers and are small in size compared to the somatic cells) most likely get discarded while processing as discussed recently [19]. Rather than using the whole ovary for Fluorescent-Activated (FACS) or Magnetic-Activated Cell Sorting (MACS) to isolate OSCs, we manually scrape the ovarian surface of higher mammals (sheep, rabbit, marmoset, human) and partial enzymatic digestion of mice ovaries to obtain OSE cells in a culture dish taking care to always spin at a speed of 1000 g which is crucial to pellet VSELs [19]. Cell suspension thus obtained is used to characterize OSCs by various techniques like flow cytometry, Hematoxylin and Eosin (H&E), immuno-staining and RT-PCR. Apart from DDX-4, one could also use markers like OCT-4 and SSEA-4 to study stem cells in OSE. Guo et al. [30] have recently provided strong evidence in support of active germ cells in adult mouse ovary by using Oct-4 transgenic mouse as study model. They detected persistent DNA reduplication, mitosis and entry into meiosis and progression to primordial follicle in young adult mice ovaries over a period of 4 months. Results were confirmed by immuno-expression, RT-PCR and BrdU uptake studies. Moreover, Zhang and Wu [31] established a cell line of double positive (DDX4 and EdU) cells from young adult 21 days old Ddx4-Cre; mT/mG mouse ovaries and these cells were able to produce an offspring on transplanting in sterilized recipients after mating.

The present study was undertaken to gain further insights into adult sheep OSCs biology and also to delineate how these stem cells may function in vivo. Sheep ovaries were preferred for the studies as they are bigger in size, more close to human ovaries and available in plenty from a slaughter house. We have generated evidence in support of presence of two populations of stem cells including VSELs and OSCs. Ovarian stem cells were further studied for the expression of pluripotent/progenitor stem cell markers and FSHR and direct effect of FSH was studied on the stem cells in vitro. We also studied differential expression of OCT-4 and NUMB to delineate whether the primitive VSELs undergo ACD to self-renew and give rise to OSCs. Besides, sheep ovarian cortical tissue sections were studied to detect stem cells and to gather evidence in support of postnatal neo-oogenesis and primordial follicle assembly in situ.

## Methods

The study was approved by Institute Committee for Stem Cell research (ICSCR) and Animal Ethics (IAEC) committee. Sheep ovaries were transported from a local slaughter house in 0.9% normal saline containing Penicillin 100 U/mL and Streptomycin 100 μg/mL (Invitrogen, USA) at room temperature (RT) and used for various studies.

### Details of various experiments undertaken in the present study

Sheep ovary is big in size, tough and fibrous compared to mouse ovary and can be easily held with forceps for scraping of OSE cells. The cortex does not get damaged and the chances of presence of contaminating granulosa cells are minimal. RT-PCR studies were undertaken to detect granulosa cells specific markers Amh and Gata-4 in the scraped OSE cells. Once it was ensured that the scraped OSE cells (enriched for stem cells) were not contaminated by granulosa cells, 3 major experiments were undertaken as mentioned below.

A. *Characterization of stem cells in sheep OSE*: The stem cells in the OSE were characterized by immuno-phenotyping studies wherein OCT-4 positive stem cells were enumerated by using tagged and untagged OCT-4 antibody. Co-expression of FSHR and OCT-4 was also studied. Expression of OCT-4, SSEA-4 and FSHR on the stem cells was studied by immunofluorescence studies. RT-PCR detection for transcripts specific for stem/progenitor cells and their proliferation was carried out using primers specific for Oct-4A, Sox-2, Oct-4, Vasa, Stat3 and Pcna. Antibody used to study OCT-4 is able to detect both the alternatively spliced OCT-4 isoforms including OCT-4A in the nucleus of VSELs and OCT-4B in the cytoplasm of the OSCs as described earlier also [15].
B. *Effect of FSH treatment on sheep ovarian stem cells* in vitro*:* OSE cells were cultured for 24 h in presence and absence of FSH (rFSH, Gonal F, Merck Serono, Switzerland). The epithelial cells get attached to the surface of the culture dish whereas stem cells remain non-adherent. Cultured cells were used to make smears to study expression of OCT-4, SSEA-4 and FSHR and for RNA extraction to study differential effect of FSH on Oct-4A, Sox-2, Oct-4, Vasa, Stat-3 and Pcna by qRT-PCR. Although Stat-3 is not a specific stem cell marker but its expression in OSE reflects presence of proliferating stem cells [32]. Dividing cells of unequal sizes suggestive of ACD were observed after FSH treatment and were studied for the co-expression of NUMB and OCT-4. Nuclear OCT-4 is a stem cell marker whereas NUMB was used to distinguish stem/progenitor cells. NUMB is known to suppress Notch signaling essential for maintaining undifferentiated stem cells [33]. During ACD, whereas the other smaller cell retains stem cell state and expresses nuclear OCT-4A, the bigger progenitors is expected to express NUMB and should be negative for nuclear OCT-4A. Thus during ACD in the ovarian stem cells, it is expected that the smaller VSEL will express nuclear OCT-4A and the slightly bigger OSC will express NUMB. Similar ACD has been reported in testicular [17] and bone marrow [21] stem cells. Ganguly et al. [21] recently reported differential expression of OCT-4 and NUMB during ACD in mouse bone marrow stem cells.
C. *Studies on sheep ovarian sections*: Ovarian sections were used to study histology and expression of PCNA, OCT-4 and FSHR. This study helped us to gather evidence how stem cells may function in vivo in adult ovary.

### Details of various methods used in the present study

Few ovaries were fixed in 10% neutral buffered formalin (NBF) at 4 °C for histological studies and immuno-histochemistry. Ovaries were also used to manually scrape OSE cells used for various studies using methods described in details below. Additional file 1: Tables S1 and S2 show details of antibodies and primers used for the study.

#### Isolation of OSE cells

Ovaries were rinsed 3–5 times with calcium and magnesium free Dulbecco’s phosphate-buffered saline (DPBS; Invitrogen) containing antibiotics (1X PenStrep). Surrounding extraneous tissue was removed without disturbing the OSE layer. Ovaries were placed in DMEM/F12 high-glucose (Sigma-Aldrich, USA) with 1X antibiotics and their surface was gently scraped with the help of a sterile blunt cell scraper to release the OSE cells as described earlier [10, 11]. These OSE cells were filtered through 40 μm sieve (BD Bio Sciences, USA) and were washed using 1X PBS by spinning cells suspension at 1000 g for 10 min at RT. Cell pellets were re-suspended in 1X PBS or plain DMEMF12 medium and used to make smears, for RNA extraction, flow cytometry and culture studies.

#### Preparation of sheep OSE cell smears

OSE cells smears were prepared on poly-L-lysine (Sigma-Aldrich,) coated slides. Cells were air dried on the glass slides, fixed with 4% paraformaldehyde (PFA; Sigma-Aldrich) for 15 min followed by 3 to 4 washes with 1XPBS. Slides were then air dried and stored at 4 °C until further use. Smears were used for H&E staining to study different cell types and also for immuno-localization studies (OCT-4, SSEA-4, FSHR & VASA).

#### Culture of OSE cells

The OSE cells were collected in DMEM/F12 medium supplemented with 10% fetal bovine serum (FBS, Invitrogen,) with antibiotics and were cultured in 5% CO_2_ incubator at 38.5 °C with or without FSH (5 IU/ml, Gonal F). Effect of FSH treatment on ovarian stem cells was studied by H&E staining, immuno-localization (OCT-4, SSEA-4, VASA, FSHR, PCNA) and qRT-PCR (Oct-4A, Sox-2, Stat-3, Total Oct-4, Vasa, Fshr1 and Fshr3) and proliferation (Pcna) specific markers with and without FSH.

#### Immuno-localization studies

Immuno-fluorescence (IF): For IF studies, the OSE smears were hydrated in 1X PBS, followed by 2 h blocking in PBS containing 10% normal goat serum (NGS) and 1% bovine serum albumin (BSA) (Sigma-Aldrich). After removing excess blocking solution, the smears were incubated overnight with primary antibody against FSH receptor (1:100), OCT-4 (1:100) and SSEA-4 (1:100) at 4 °C. Permeabilization with 0.3% Triton X (Sigma-Aldrich) was done for 5 min to study nuclear and cytoplasmic expression of OCT-4 prior to incubating with primary antibody. Next day the slides were brought to RT and washed three times (5 mins each wash) with phosphate buffered saline (PBS) to remove excess unbound antibody. Later the cells were incubated with respective secondary antibody Alexa flour (Molecular Probes, Invitrogen) 488 or 568 (1:1000) for 2 h at RT. The smears were then counterstained with propidium iodide (PI, Sigma-Aldrich; 5 mg/ml) for 2 min or 4′ 6-diamidino-2-phenylindole (DAPI, Sigma-Aldrich; 1.47 μM) for 20 min. Finally, smears were washed with PBS and mounted using Vectashield and stored at 4 °C till viewing. The slides were scanned under laser scanning confocal fluorescent microscope (LSM 510-META, ZEISS, Germany) and representative images were photographed.

Immuno-histochemistry (IHC): Briefly, the paraffin embedded ovarian tissue sections were deparaffinized and incubated in xylene for 30 mins. After air drying, the slides were incubated with 3% hydrogen peroxide (Qualigens, India) in 100% methanol for 1 h in dark after which the sections were gradually hydrated in descending series of methanol to tap water for 5 min each. Antigen retrieval was done by immersing the slides in boiling sodium citrate (SSC, Sigma-Aldrich) buffer at pH 6 for 5 mins. After cooling, the slides were washed with 1X PBS buffer for 5 min. Permeabilization step was carried out by 0.3% TritonX-100 in PBS buffer for 5–7 min to studyOCT-4 and PCNA expression. After blocking for 2 h with 2% BSA along with 5% normal goat serum (NGS) for OCT-4 and FSHR antibodies and with 5% normal horse serum (NHS) for PCNA antibody, the slides were incubated overnight at 4 °C with OCT-4, FSHR and PCNA antibodies. Next day slides were washed 3 times with PBS (5 min each wash) and then incubated with respective biotinylated secondary antibody for 30 min followed by avidin biotin complex formation step for 30 min (Vectastain Elite ABC kit, Vector Laboratories Inc., USA), 3 washes with PBS and then color reaction was done using diaminobenzidene (Biogenex, USA). The slides were then counterstained with Hematoxylin, dehydrated, cover slipped and sealed using nail polish. Representative areas were photographed under Nikon 90i microscope (Nikon, Japan).

Immuno-cytochemistry (ICC): For ICC, after the blocking step in dark at room temperature using 3% hydrogen peroxide in PBS for 30 min, the smears were subjected to antigen retrieval by incubating with sodium citrate buffer (10 mM sodium citrate), pH 6.0, at high power for 5 min in a microwave oven. This was followed by permeabilization step with 0.3% Triton-X 100 for 10 min. Further procedure for immuno-localization was similar to that described for IHC above.

Primary antibody was omitted in the negative controls for various experiments. To further demonstrate the specificity of FSHR expression on the stem cells, peptide against which FSHR antibody was raised was incubated overnight along with the antibody at 4 °C and used as another negative control for various experiments.

##### Flow cytometry studies

Immuno-phenotyping studies using sheep OSE cells have earlier [14] shown the presence of two distinct populations of OCT-4 positive stem cells (Additional file 1: Figure S1). In the present study further immuno-phenotyping studies were carried to evaluate OCT-4, and FSHR positive cells in OSE. Briefly, single cells suspension of OSE cells were prepared as described above and were fixed in 2% PFA for 15 min. The cells were washed twice with PBS and then permeabilized for 5 to 10 min using 0.1% NP40 solution (Sigma) in PBS for studying OCT-4 expression whereas no permeabilization was done to study cell surface expression of FSHR. Indirect labeling with primary antibodies (OCT-4, FSHR) was performed by incubating cells with them for 60 min at 4 °C followed by secondary antibodies (Alexaflour goat anti-mouse IgG 568 or IgG 488) for 45 min (Molecular Probes, Invitrogen). Direct labeling was done for OCT-4 PerCP-Cy tagged antibody (BD, USA) for 45 min at 4 °C. Following three washes with PBS, the events were acquired and analyzed using FACS DIVA software and FCS Express 4 software (De Novo software). Calibration beads of size 2 to 15 μm were used according to the manufacturer’s instructions (Life Technologies, USA) as reference for selecting cells ranging from 2 to 6 μm. Care was taken to spin the cells at 1000 g while pelleting the cells throughout the experiment since the stem cells do not pellet down at 200-250 g [14, 19].

#### RNA extraction, RT-PCR and qRT-PCR studies

RNA was extracted from OSE cells using TRIZOL (Invitrogen) by standard protocol followed by DNase I (Amersham Biosciences, USA) treatment at 37 °C for 30 min to remove any genomic DNA contamination. Reverse transcription of cDNA was performed using iScript cDNA synthesis kit (Bio-Rad, USA) according to the manufacturer’s instructions. Briefly, RNA was incubated with 5X iScript reaction mix and iScript reverse transcriptase mix. The reaction was carried out in G-STORM thermocycler (Gene Technologies, UK). The reaction mix was first incubated at 25 °C for 5 min, then at 42 °C for 30 min and finally at 85 °C for 5 min.

RT-PCR was carried out to detect transcripts specific for stem cells (Oct-4A, Sox-2, Stat-3, Total Oct-4, Vasa) and granulosa cells (Amh, Gata-4) along with Gapdh as housekeeping transcript. Briefly, the cDNA was incubated with Taq DNA polymerase, 1.5 mm MgCl2 and 10 mM deoxynucleotide triphosphates (Fermentas Life Sciences, USA) with specific primers (10 pmol) in a 25 μl reaction volume using G STORM thermocycler. PCR program included initial denaturation at 95 °C for 3 min, followed by denaturation for 1 min, annealing at different temperatures (Additional file 1: Table S2) and final elongation at 72 °C for 50s. for 35–40 cycles. The RT-PCR products were visualized by electrophoresis on 2% agarose gel.

The expression levels of various transcripts were also estimated by CFX96 real-time PCR system (Bio-Rad Laboratories, California, USA) using SYBR Green chemistry (Bio-Rad). Gapdh was used as housekeeping in all the experiments. The amplification conditions were described earlier: initial denaturation at 94 °C for 3 min followed by 40 cycles comprising of denaturation at 94 °C for 30 s, primer annealing at specific temperature (Additional file 1: Table S2) for 30 s, and extension at 72 °C for 30 s. The final extension was carried out for 5 mins at 72 °C. The fluorescence emitted at each cycle was captured during the extension step of each cycle. The homogeneity of the PCR amplicons was verified by running the products on 2% agarose gels. Each gene amplification was carried out in duplicate. The fold change expression of each transcript was studied from three different experimental replicates.

## Results

Initially it was ensured that only ovary surface epithelial cells and stem cells were collected by the method of gentle scraping of ovary surface (Additional file 1: Figure S2) and there was no contamination by the granulosa cells. For this, RT-PCR for 2 granulosa cell markers (Amh, Gata-4) was carried out and these transcripts were not detected in the OSE cells (Additional file 1: Figure S3).

### Characterization of stem cells in sheep ovary surface epithelium

#### Immuno-phenotyping studies

**Fig. 1:**
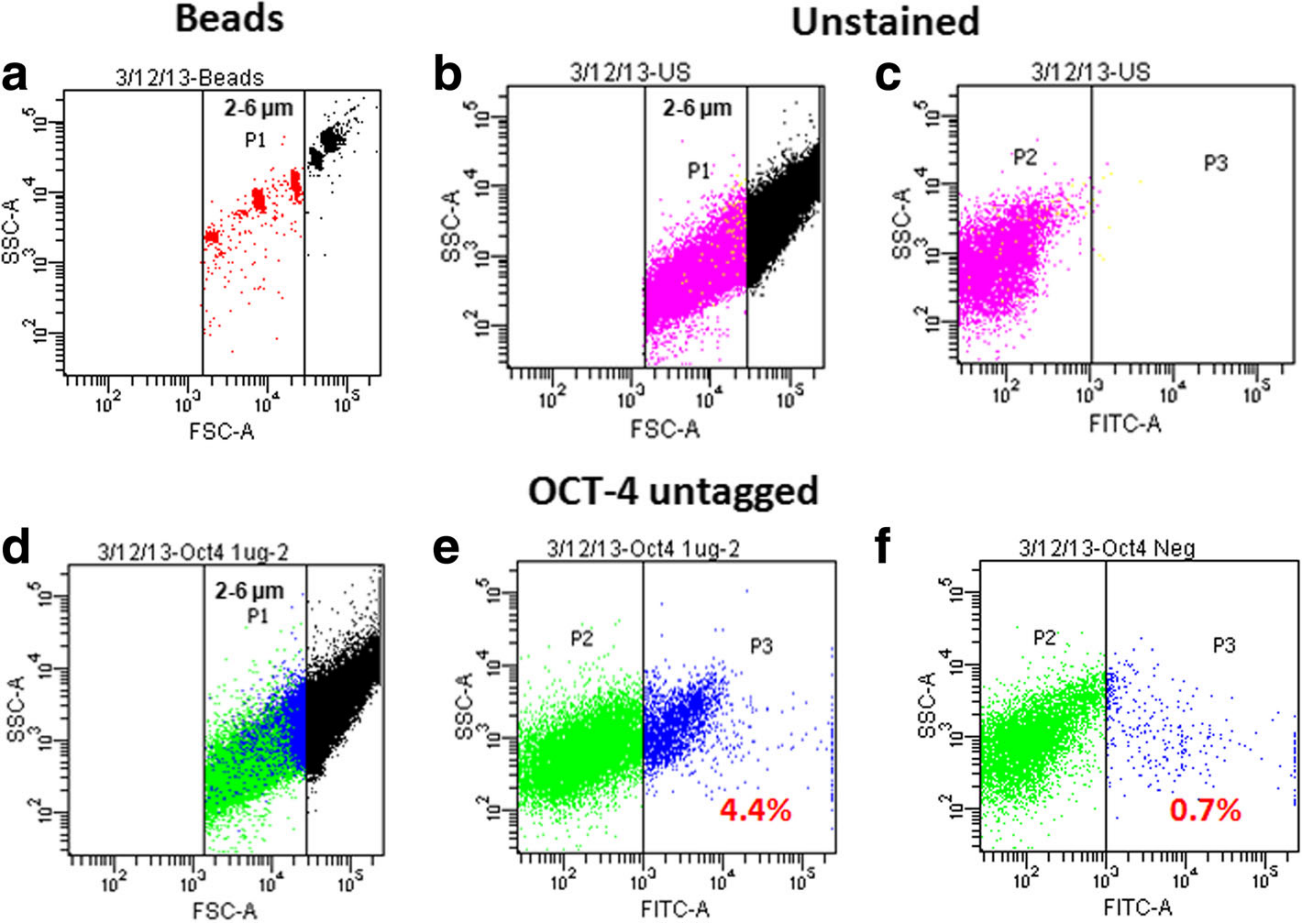
Immuno-phenotyping studies on stem cells in sheep ovarian surface epithelial (OSE) cells. Immunophenotyping analysis for OCT-4 was carried out on sheep OSE cells (**a**) using standard calibration beads of size ranging from 2-6 μm gated as P1 (**b**) sheep OSE cellsin the size range of 2-6 μm size representing P1 population (**c**) represents unstained cells for FITC gated as P3 population. (**d**) Immuno-phenotyping analysis for stem cells in the size range of 2-6 μm with (**e**) 4.4% cells expressing (untagged) OCT-4 antibody labelled with Alexafluor 488 whereas (**f**) represents percentage of cell population negative for OCT-4 using only Alexafluor 488 without primary antibody serving as negative control

**Fig. 2:**
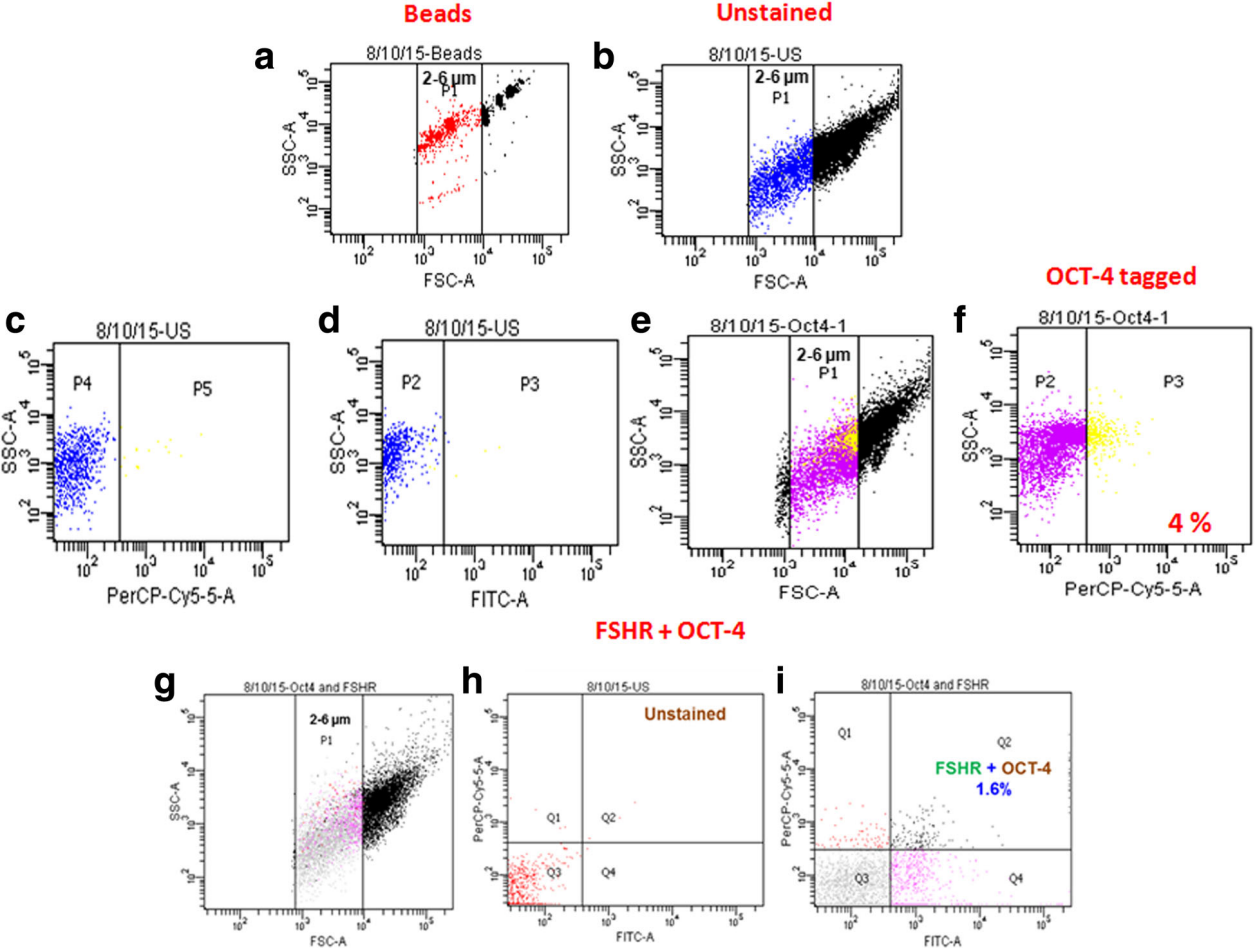
Immuno-phenotyping studies for OCT-4 and co-expression of OCT-4 and FSHR. Immuno-phenotyping analysis for OCT-4 and FSHR + OCT-4 was carried out on sheep OSE cells (gating strategy similar to that described in Fig. 1) (**a**) represents standard calibration beads of size ranging from 2–6 μm gated as P1, (**b**) sheep OSE cellsin the size range of 2-6 μm size representing P1 population (**c**, **d**) unstained cells for both PerCP-Cy5-5A and FITC gated as P5 and P3 populations respectively. (**e**) Immuno-phenotyping analysis for stem cells in the size range of 2-6 μm with (**f**) 4% cells expressing PerCP-Cy5-5A tagged OCT-4 antibody and (**g**) sheep OSE cells in the size range of 2–6 μm size representing P1 population (**h**) represents unstained cells for both PerCP-Cy5-5A and FITC (**i**) 4% of cells in the size range of 2–6 μm were OCT-4 positive and 6.5% expressed FSHR of which 1.6% of cells were also positive for OCT-4

Immuno-phenotyping studies were undertaken to enumerate cells in the size range of 2–6 μm expressing OCT-4 using untagged (Fig. 1) and tagged (Fig. 2) antibodies. 4.4% of cells in the size range of 2–6 μm expressed OCT-4 by indirect staining method using untagged antibody. 4% of cells were found positive using directly tagged OCT-4 antibody. Furthermore, 6.5% of OSE cells in the size range of 2–6 μm expressed FSHR and 1.2% of these cells co-expressed FSHR and OCT-4(Fig. 2).

#### Immuno-fluorescence studies

**Fig. 3:**
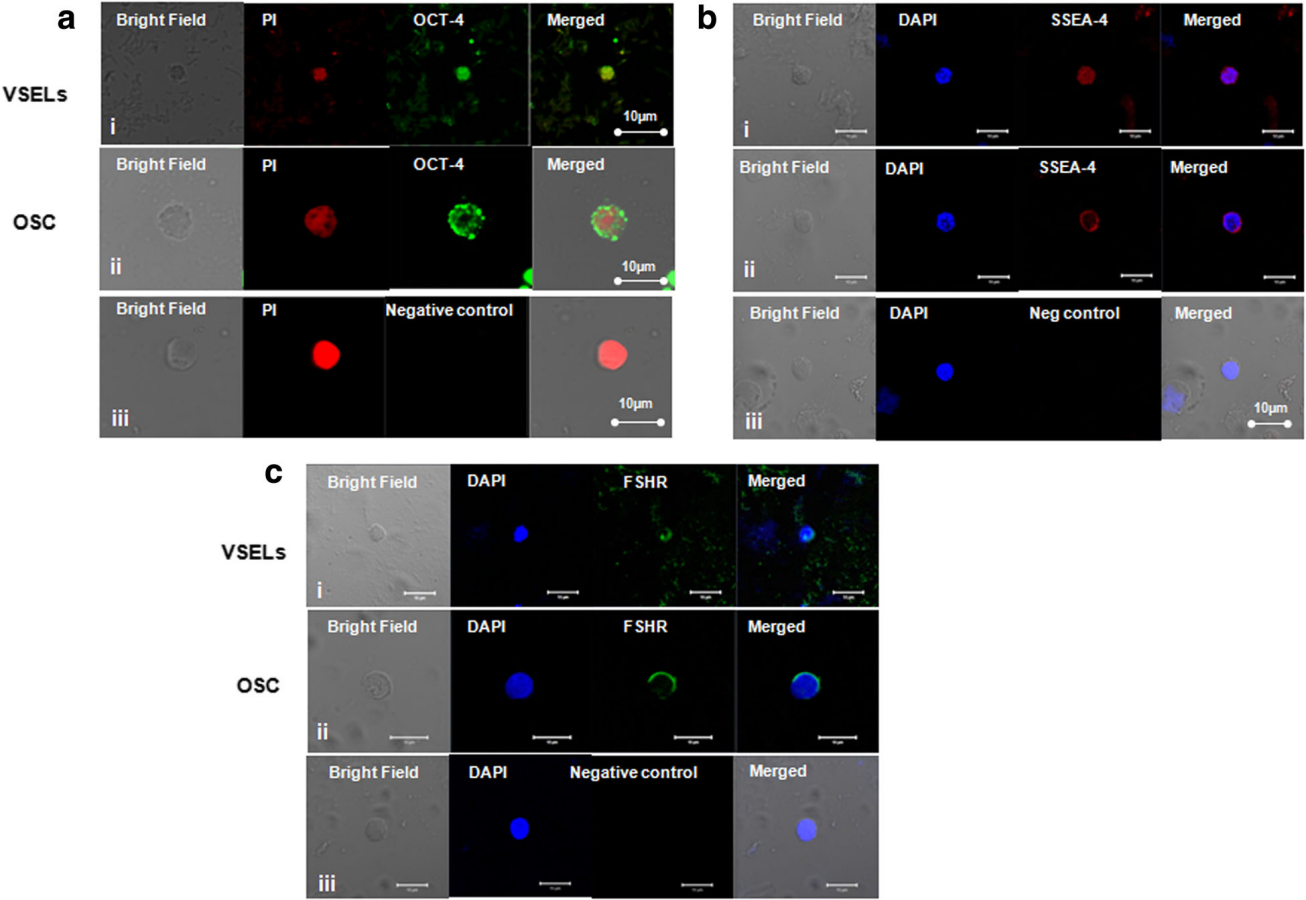
OCT-4, SSEA-4 and FSHR expression on ovarian stem cells. Cells with nuclear OCT-4 (Ai) and surface SSEA-4 (Bi) and FSHR (Ci) represent pluripotent VSELs, and slightly bigger cells with cytoplasmic OCT-4 (Aii) minimal surface SSEA-4(Bii) and FSHR (Cii) represent progenitor OSCs. Negative control by omission of primary antibody showed no staining (**a**, **b** & **c** iii)

**Fig. 4:**
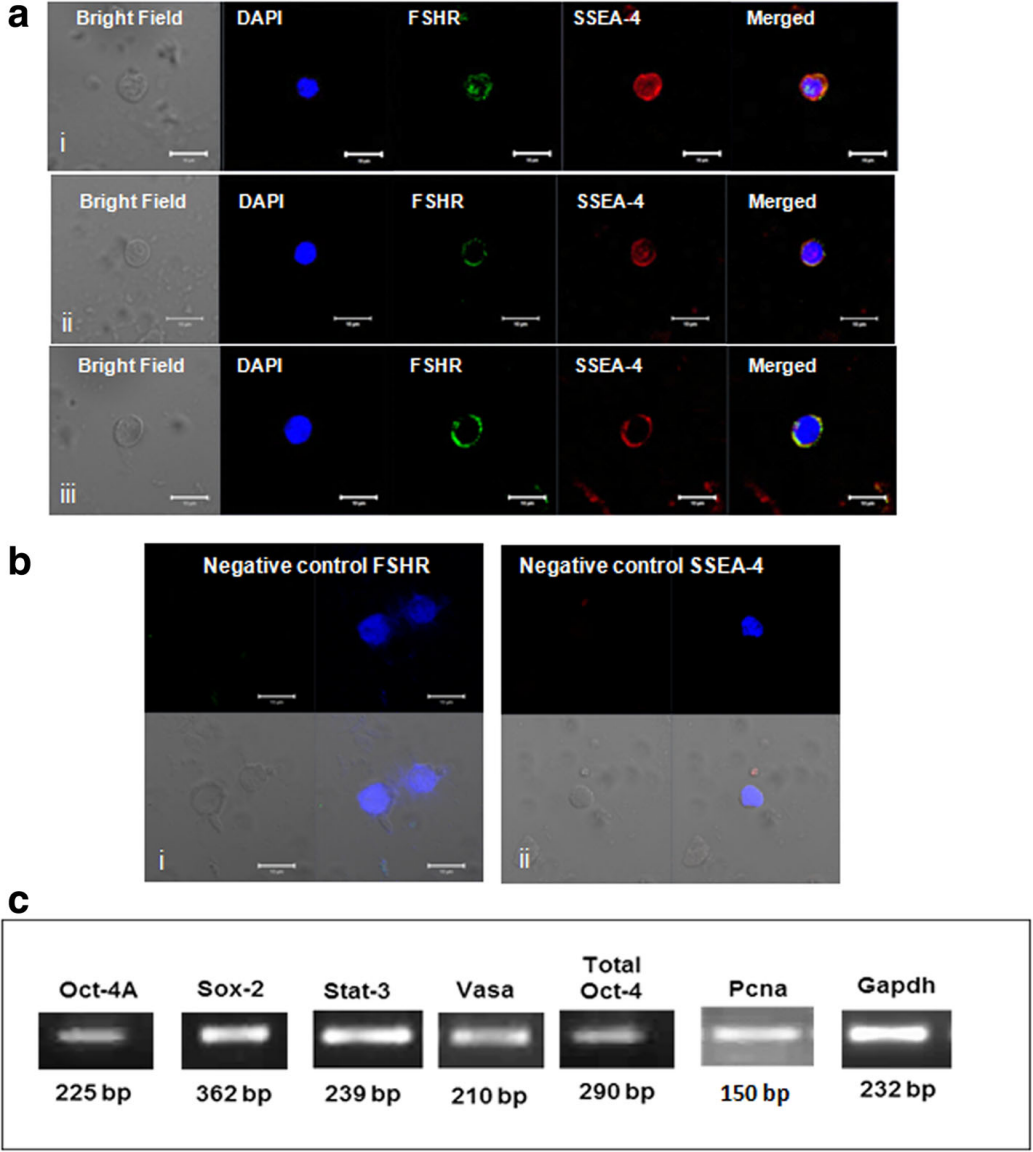
Co-expression of FSHR and SSEA-4 on ovarian stem cells. **a** Co-expression of SSEA-4 and FSHR on stem cells (**b**) Negative control by omission of primary antibody for both FSHR and SSEA-4 (**c**) RT-PCR results on manually scraped OSE cells showing amplification of pluripotent (Oct-4A, Sox-2, Stat-3), progenitor (Total Oct-4) and germ (Vasa) cells markers along with marker for proliferation (Pcna) and housing keeping gene (Gapdh) at transcript level

OCT-4, SSEA-4 and FSHR expression was studied on stem cells by confocal microscopy (Fig. 3). Nuclear OCT-4 was found localized in small sized VSELs (Fig. 4Ai) and cytoplasmic OCT-4 in the slightly bigger OSCs (Fig. 3Aii) with PI as nuclear stain and negative control (lacking primary antibody) showed no IF signals (Fig. 3Aiii). Similar staining pattern was observed for SSEA-4 in VSELs (Fig. 3Bi) and OSCs (Fig. 3Bii) with DAPI as nuclear stain and absent signals in negative control (Fig. 3Biii). FSH receptors were localized on the surface of both stem cell populations (Fig. 3c) with DAPI as nuclear stain. Stem cells were found to co-express SSEA-4 and FSHR (Fig. 4a) whereas Fig. 4b is negative control. Additional data showing expression of FSHR and co-expression of OCT-4/SSEA-4 and FSHR on the ovarian stem cells is provided in the supplement including a Z-stack of OCT-4 expression on stem cells (Additional file 1: Figures S4–S6).

#### RT-PCR analysis

RT-PCR analysis on the manually scraped OSE cells showed the presence of transcripts specific for stem/progenitor cells including Oct-4A, Sox-2, Oct-4, Vasa, Stat3 and Pcna (Fig. 4c), thus confirming the presence of stem/progenitor cells in OSE.

To conclude from this section, scraped OSE cells comprise epithelial cells, stem cells and few red blood cells and are not contaminated by granulosa cells and thus FSHR expression is on the stem cells. Immuno-phenotyping studies confirm that stem cells in the size range of 2–6 um exist in OSE that express OCT-4 and a sub-population of these stem cells also express FSHR. OCT-4 and FSHR expression was also confirmed by confocal microscopy and at mRNA level by RT-PCR. FSHR expression on OSCs provides a paradigm shift in current understanding of FSH action. We have earlier reported similar expression of FSHR on testicular stem cells by using 3 different antibodies [17] and thus use of a single antibody in the present study sufficed to show similar presence of FSHR on ovarian stem cells. FSHR detection by qRT-PCR and in situ hybridization using specific oligoprobes has been reported earlier on ovarian stem cells [11]. Similar activation of mouse [34] and human [35] OSCs by FSH has also been reported earlier.

### Effect of FSH treatment on sheep ovarian stem cells in vitro

**Fig. 5:**
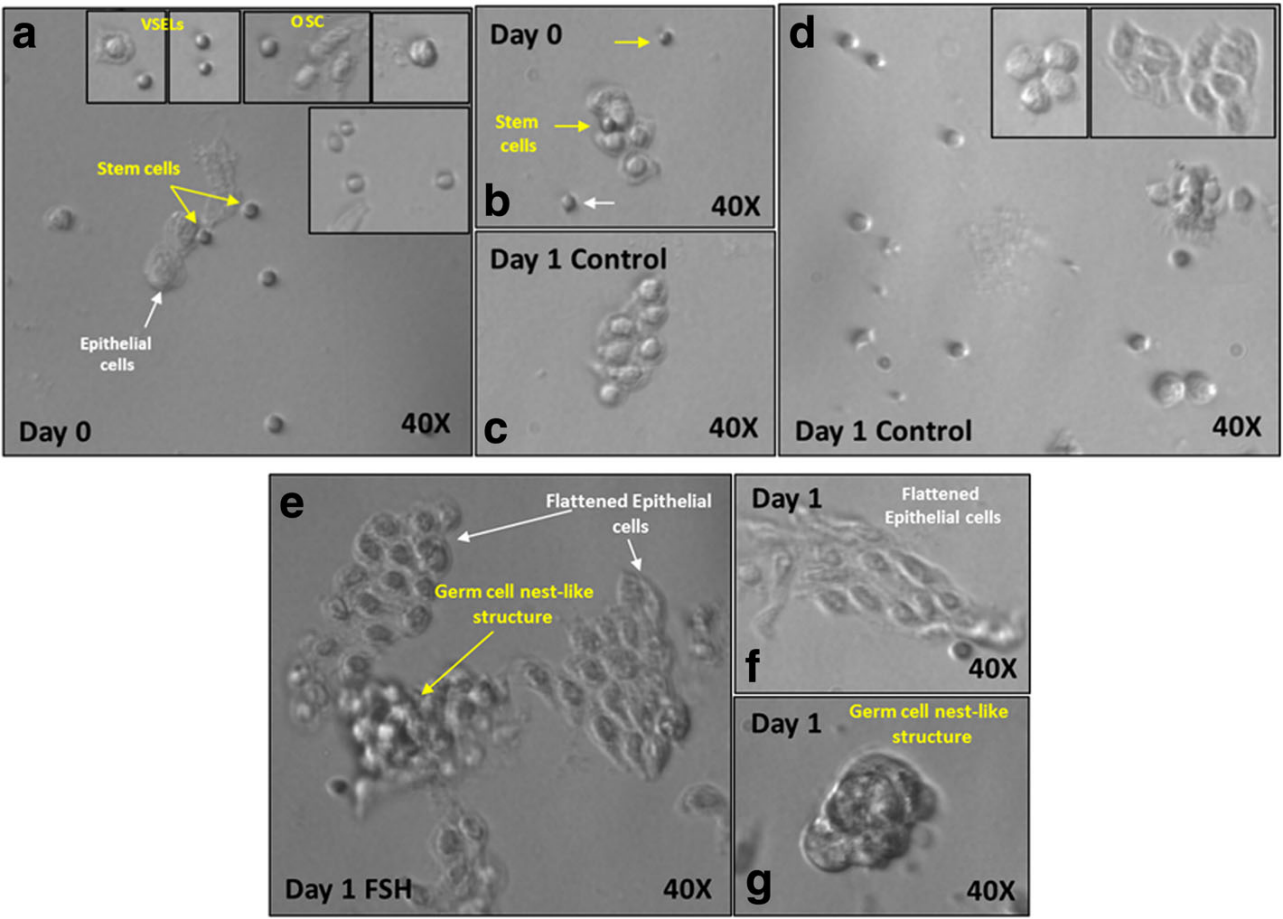
In vitro culture of manually scraped sheep ovary surface epithelial (OSE) cells in presence of FSH. **a**-**b** OSE cells in initial culture show presence of different cell types including large epithelial cells (white arrow), small spherical VSELs and slightly larger OSCs (yellow arrow). Stem cells are observed in close association with large epithelial cells. **c**-**d** Untreated (without FSH) OSE cells after 24 h of culture show minimal effect on stem cells **e**-**g** FSH treatment for 24 h resulted in distinct changes in OSE culture. Spherical stem cells appeared to increase in numbers and formed small spheres in close association with epithelial cells bed. **f** Epithelial cells became flat and were attached to bottom of the culture dish. **g** Cluster of stem cells formed by incomplete cytokinesis (clonal expansion) resembled germ cell nest-like structures

Different cell types were observed when scraped OSE were observed under a microscope (D0) including large flat epithelial cells and spherical stem cells of two distinct sizes including VSELs and OSCs (Fig. 5a & b). After 24 h of culture, epithelial cells became flat, undergo epithelial-mesenchymal transition into fibroblasts and were attached to the bottom of the culture dish (Fig. 5c) whereas spherical stem cells were found singly or in close proximity to the somatic bed in the culture (Fig. 5d). Addition of FSH in the culture medium for 24 h had a prominent effect on the stem cells which were found to exist in small clusters (Fig. 5e-g) compared to untreated control where the spherical cells were seen singly (Fig. 5c & d). These clusters of progenitor stem cells result due to incomplete cytokinesis and resemble germ cell nests (Fig. 5g) reported in fetal ovaries and as reported earlier by our group [12]. More number of germ cell clusters were observed after FSH treatment compared to untreated control. Evidently, FSH treatment stimulated the stem cells to undergo proliferation and clonal expansion to form germ cell nests (Fig. 5g) which exist on top of the flattened somatic cells bed (Fig. 5e).

**Fig. 6:**
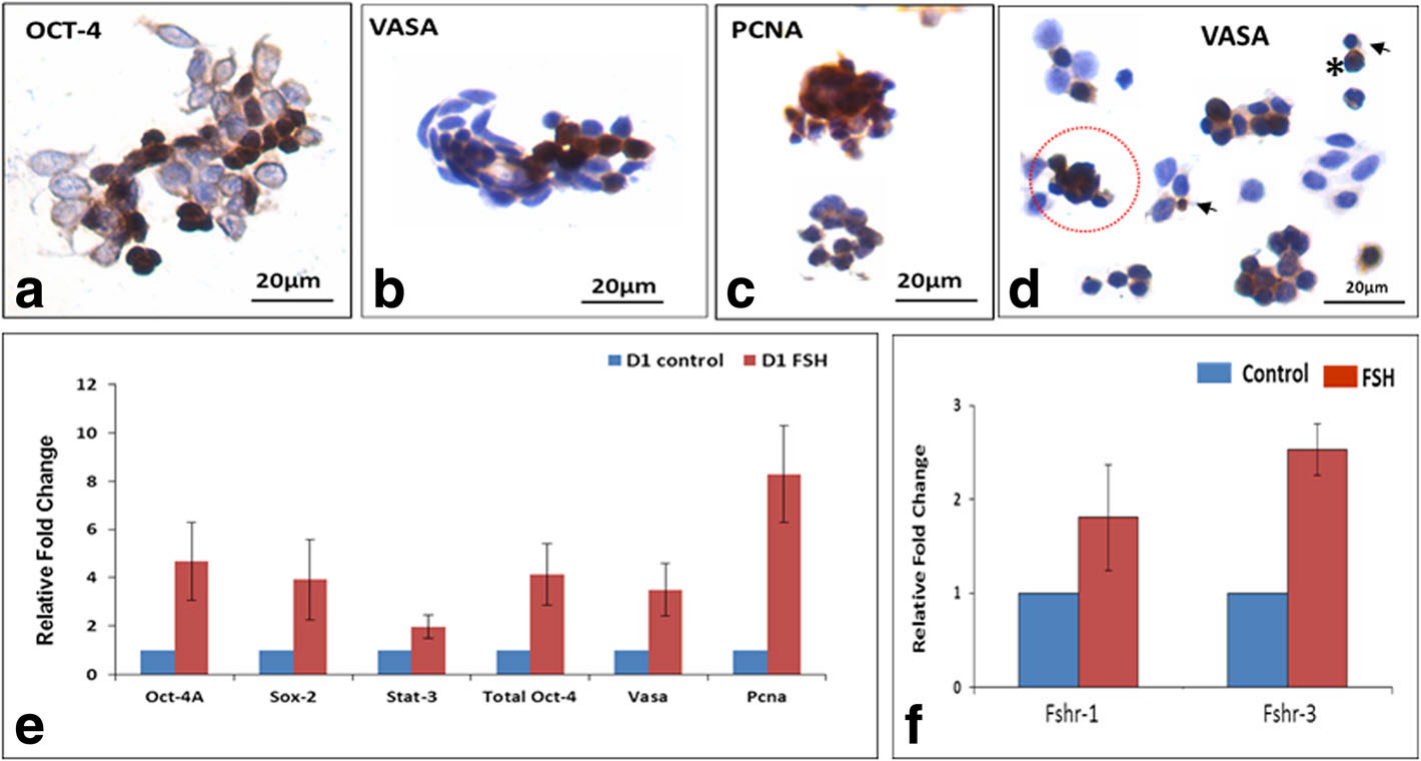
Characterization of germ cell nest-like structures obtained after FSH treatment. These structures with incomplete cytokinesis were observed to be positive for (**a**) OCT-4 (**b** & **d**) VASA and (**c**) PCNA suggesting stem cells proliferation and clonal expansion in response to FSH treatment. Both small VSELs (arrow) and slightly bigger OSCs (asterix), asymmetric cell division and clonal expansion (circled) were clearly visualized. Note that the epithelial cells were distinctly negative for these markers. Similar structures have been earlier reported also with detailed characterization to show that they indeed represent incomplete cytokinesis and not cell aggregates [6, 7]. **e** qRT-PCR results on OSE cells showed increased expression of VSELs (Oct-4A, Sox-2, Stat-3), OSCs (Oct-4 and Vasa) and proliferation (Pcna) specific transcripts after 24 h of FSH treatment (red bars) compared to untreated controls (blue bars). Results are representative of three different experiments and error bars represent standard error **f** Transcript for alternately spliced FSHR isoform, Fshr3 appears to be more up-regulated after FSH treatment compared to the canonical isoform Fshr1 in agreement with earlier results [6, 12]

**Fig. 7:**
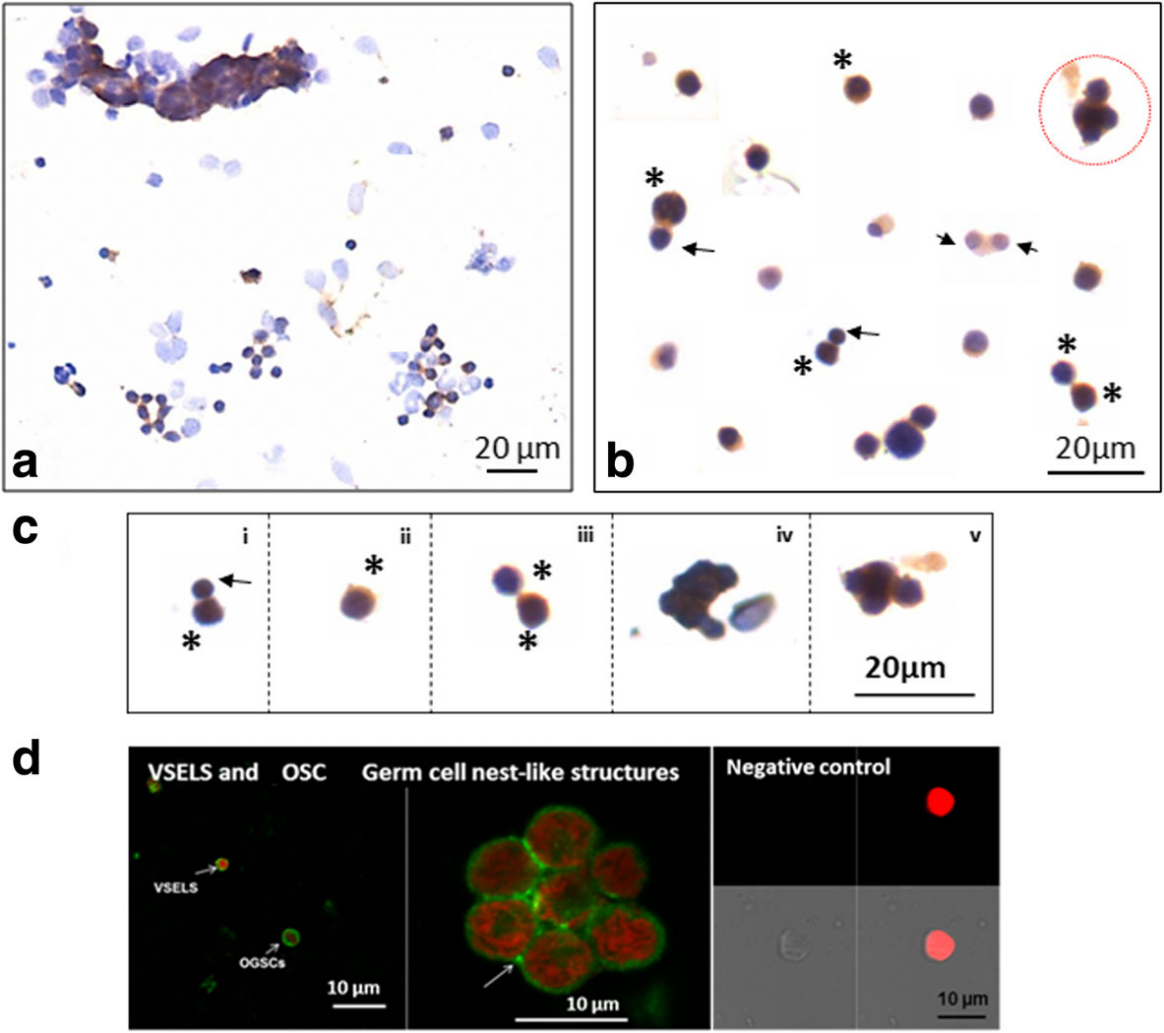
FSHR expression on cells obtained by scraping sheep ovary surface after FSH treatment in vitro. **a** Low magnification showing epithelial cells and stem cells in close vicinity with FSHR expression only on the stem cells. **b** Various fields were photographed to study stem cells division. Two distinct size of FSHR positive stem cells were visualized including slightly small VSELs (arrow) and bigger OSCs (asterix). Both asymmetric and symmetric cell divisions and germ cell nest-like structures (circled) were clearly visualized. Please note that both (**a**) and (**b**) are actually composites prepared by putting together various fields as these cells are spread far apart on the slides. **c** Stem cells are linearly arranged to understand their biology. (i) Small sized VSEL undergoes asymmetric cell division to give rise to slightly bigger OSCs which (ii) OSC (iii) undergo symmetric cell division (iv-v) and clonal expansion with incomplete cytokinesis to form a germ cell nest-like structure. Similar germ cell nest-like structures in adult ovary have been reported earlier also [6, 7]. **d** Representative confocal images showing FSHR expression on VSELs/OSCs/germ cell nest-like structure and negative control

Spherical stem cells present singly or in small clusters expressed OCT-4, VASA and PCNA (Fig. 6a-d) whereas the somatic cells remained distinctly negative. qRT-PCR analysis of OSE cells after 24 h of FSH treatment compared to untreated controls showed an increased expression of stem/germ cell (Oct-4A, Sox-2, Stat-3, Total OCT-4 and Vasa) along with proliferation (Pcna) specific transcripts suggesting a stimulatory effect of FSH on the stem cells (Fig. 6e). Moreover, alternately spliced Fshr3 was markedly up regulated by FSH treatment compared to Fshr1 after 24 h of FSH treatment (Fig. 6f). Further ICC studies confirmed FSHR expression on the stem cells (Fig. 7a-b). Both symmetrical (daughter cells of similar size) and asymmetrical (daughter cells of unequal size) cell divisions were observed after FSH treatment (Fig. 7c) similar to our earlier findings in testis [17] To clearly understand the heterogeneity of ovarian stem cells in OSE, various cells were arranged to understand their hierarchy (Fig. 7c) on the basis of their size. As evident, small VSELs underwent asymmetric cell division to give rise to slightly bigger progenitors OSCs which underwent symmetric cell division and clonal expansion with incomplete cytokinesis to form spheres resembling gem cells nests described in fetal ovaries. Additional file 1: Figure S7 shows different fields that were placed together to make the composite shown in Fig. 7. FSHR expression was also studied by confocal microscopy on cultured OSE cells. VSELs, OSCs and germ cell nest were found to express FSHR (Fig. 7d).

**Fig. 8:**
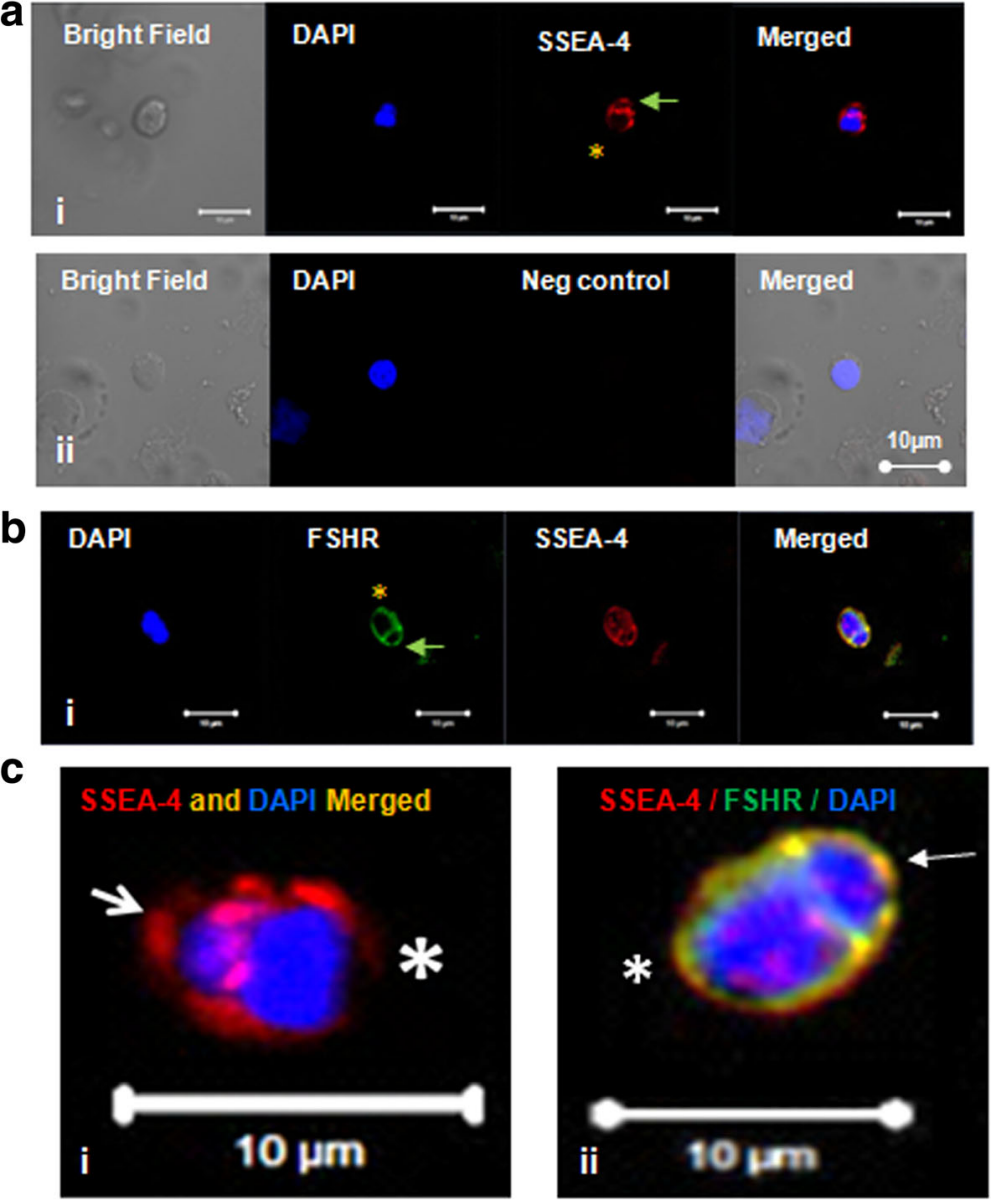
Expression of SSEA-4 and FSHR on stem cells after overnight exposure to FSH in vitro. (**A**i) SSEA-4 and DAPI positive VSEL (green arrow) undergoing asymmetric cell division and give rise to OSC (yellow asterisk). (**B**i) Co-expression of FSHR and SSEA-4 on dividing stem cells of different sizes (**c**) Magnified view of Ai and B

**Fig. 9:**
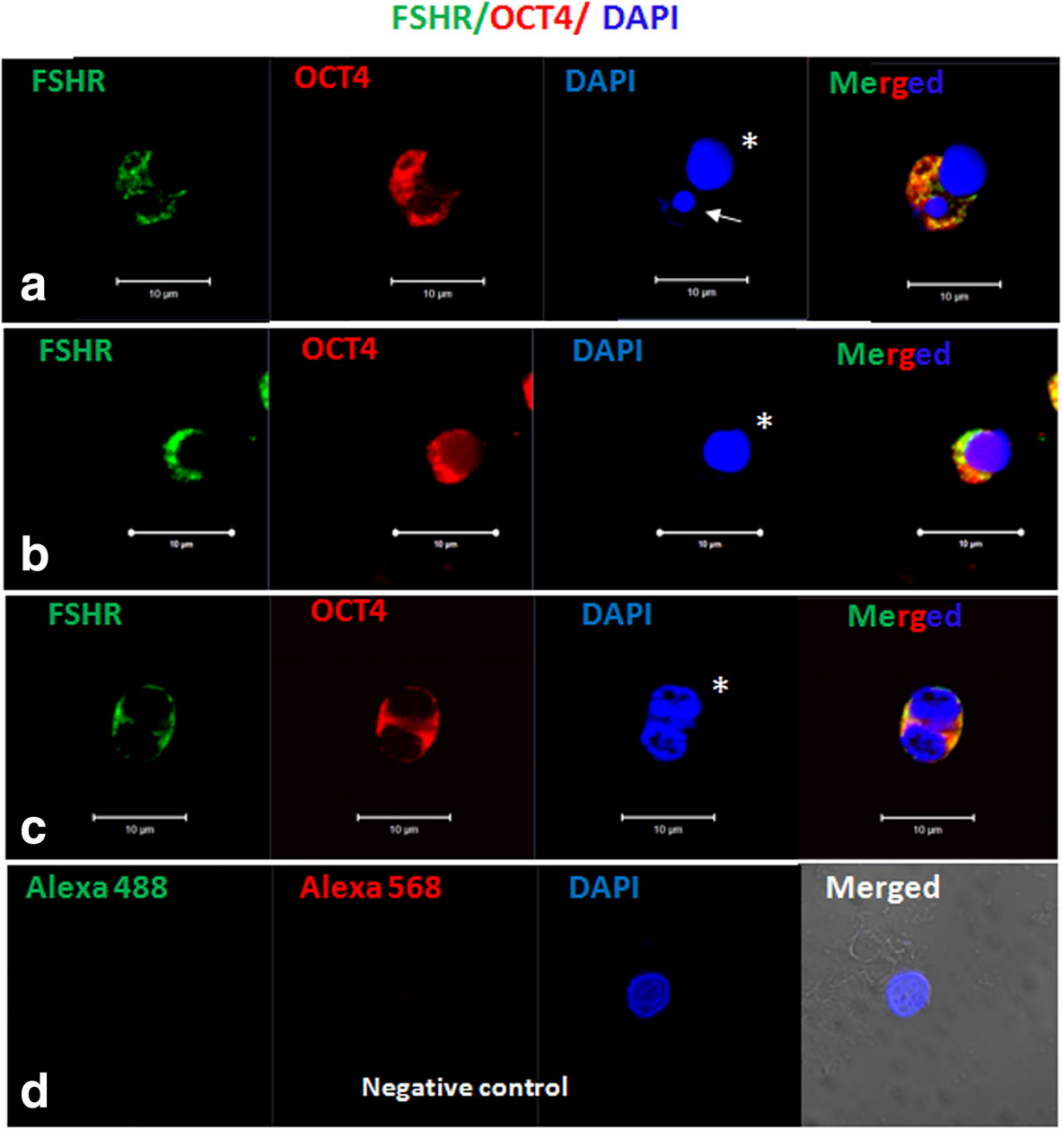
Co-expression of OCT-4 and FSHR on stem cells after overnight exposure to FSH in vitro. **a** OCT-4 (Red) and FSHR (green) positive small VSEL (arrow) undergoing asymmetric cell division and give rise to large progenitor OSC (asterisk). **b** Co-expressing FSHR and OCT-4 positive ovarian stem cells (**c**) Co-expression of FSHR and OCT-4 on dividing stem cells of same sizes representing symmetric cell division. **d** Negative control by omission of primary antibody showed no staining. Scale bar 10 μm

**Fig. 10:**
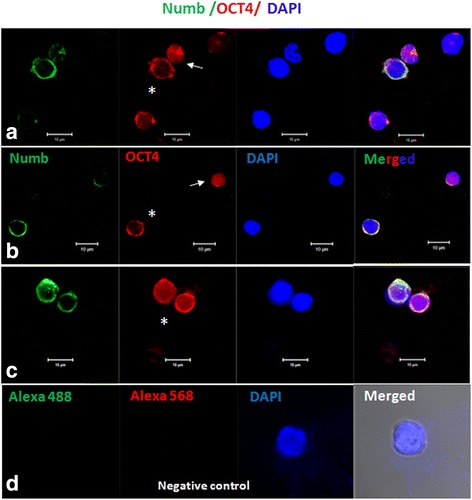
Co-expression of NUMB and OCT-4 on dividing ovarian stem cells **a** Nuclear OCT-4A positive VSEL (arrow) undergoing asymmetric cell division (ACD) to give rise to OSC (asterisk). **b** Co-expression of OCT-4A and NUMB on different sizes of stem cells shown different expression pattern for (NUMB an OCT-4A). Please note that smaller stem cells express nuclear OCT-4A (red) with minimal expression of NUMB. Whereas slightly bigger progenitor cell show OCT-4A expression in cytoplasm but not in nucleus with clear uniform distinct expression pattern for NUMB (green) in cytoplasm. **c** Co-expression of NUMB and OCT-4 on progenitor ovarian stem cells undergoing symmetric cell division (SCD). Please note progenitor cells are of equal sizes and expresses similar expression pattern for (NUMB and cytoplasmic OCT-4A). **d** Negative control by omission of primary antibody showed no staining. Cells were counterstained with DAPI. OCT-4A is a nuclear transcription factor suggesting pluripotent state whereas NUMB is expressed by, progenitor OSC and is implicated to suppress Notch signaling essential for maintaining undifferentiated stem cells. Scale bar 10 μm

ACD was also characterized by immuno-fluorescence studies. At places, stem cells (VSELs) expressing SSEA-4 were observed to possibly undergo asymmetric stem cell division giving rise to slightly bigger OSCs (Fig. 8Ai and Ci) and the dividing cells also co-expressed FSHR and SSEA-4 (Fig. 8b). It was intriguing to observe a small sized VSEL (co-expressing SSEA-4 and FSHR) to undergo asymmetric stem cell division giving rise to slightly bigger OSC (Fig. 8Ci-ii). Co-expression of FSHR and OCT-4 was also observed on dividing doublets (Fig. 9).ACD of VSELs to give rise to the OSCs was further studied by co-expression of OCT-4 and NUMB (Fig. 10).

To conclude from this section, results show that the cells obtained by scraping the ovarian surface include epithelial and stem cells. Epithelial cells (negative for OCT-4 and VASA) undergo epithelial-mesenchymal transition and attach to the culture surface in vitro. Spherical stem cells of two distinct sizes with high nucleo-cytoplasmic ratio are visualized and express OCT-4, VASA, PCNA. SSEA-1 and OCT-4 positive stem cells were found to co-express FSHR. Overnight culture of the cells in the presence of FSH resulted in increased expression of Fshr1 and Fshr3in agreement with earlier reports [11, 17]. Evidence is also generated to show that VSELs undergo asymmetric cell divisions to give rise to OSCs which undergo symmetric cell divisions and clonal expansion to form spheres similar to our recent reports on testicular [17] and bone marrow [21] stem cells. These dividing cells also showed differential expression of OCT-4 and NUMB.

### Histology and PCNA, OCT-4 & FSHR expression on sheep ovarian sections

**Fig. 11:**
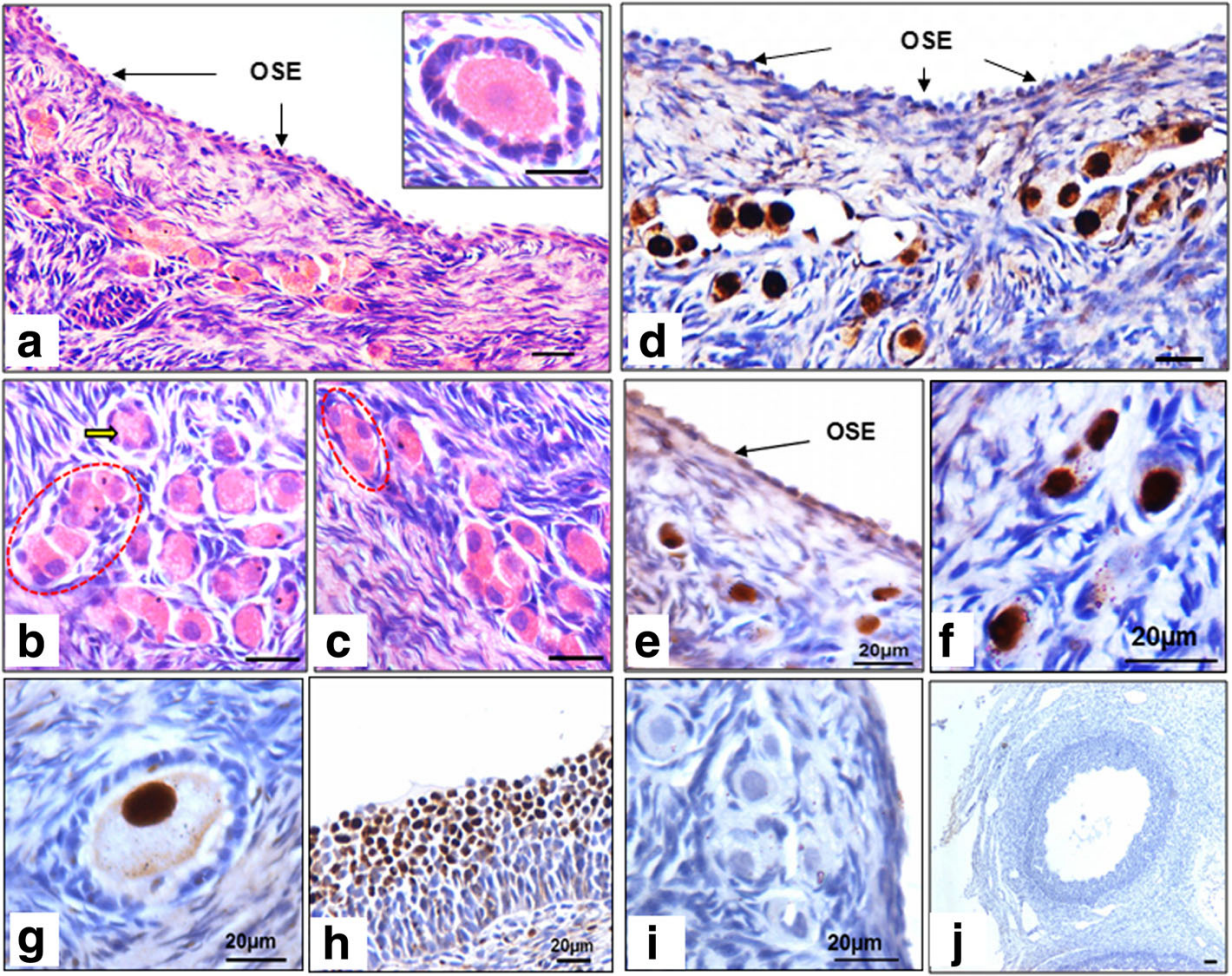
PCNA immuno-localization on sheep ovarian sections. H&E staining of ovarian sections (**a**) shows a distinct layer of OSE cells (**b**-**c**) cohort of cytoplasmically connected oocytes i.e. a germ cell nest surrounded by few pre-granulosa cells. These structures are referred to as ovigerous cords in literature (Smith et al. 2014). Few primordial and primary follicles were located in the cortical region whereas large oocytes were present in medulla region. **d**-**h** Immuno-localization with PCNA showed few OSE cells positive for PCNA, cluster of oocytes/germ cell cyst, individual primordial and primary follicles located in cortical region of ovary showed strong nuclear PCNA. Interestingly surrounding granulosa cells and stromal cells were completely negative for PCNA. Large Graffian follicle in medulla region showed weak PCNA expression in both nucleus and ooplasm of oocytes however, (**h**) surrounding granulosa cells were strongly positive for PCNA. **i** & **j** Negative control

H&E stained ovarian sections showed prominent, cuboidal, epithelial cells (Fig. 11a) lining the cortical tissue which contained large number of germ cells (Fig. 11b-c). Ovarian cortex comprised oogonial clusters devoid of surrounding granulosa cells (Fig. 11b-c) and primordial follicles surrounded by one to two layers of granulosa cells. Follicles in different stages of development including primary to secondary follicles were also observed surrounded by 2–3 layers of granulosa cells. Interestingly few cells in OSE and cohort of follicles/germ cell nest with incomplete cytokinesis in the cortex were found to express PCNA (Fig. 11d & e; Additional file 1: Figure S8). Individual oogonial cells in primordial, primary and secondary follicle oocytes were positive for PCNA whereas surrounding granulosa cells were completely negative (Fig. 11g). In contrast, granulosa cells of growing follicles expressed PCNA (Fig. 9h) whereas oocytes revealed faint PCNA expression.

**Fig. 12:**
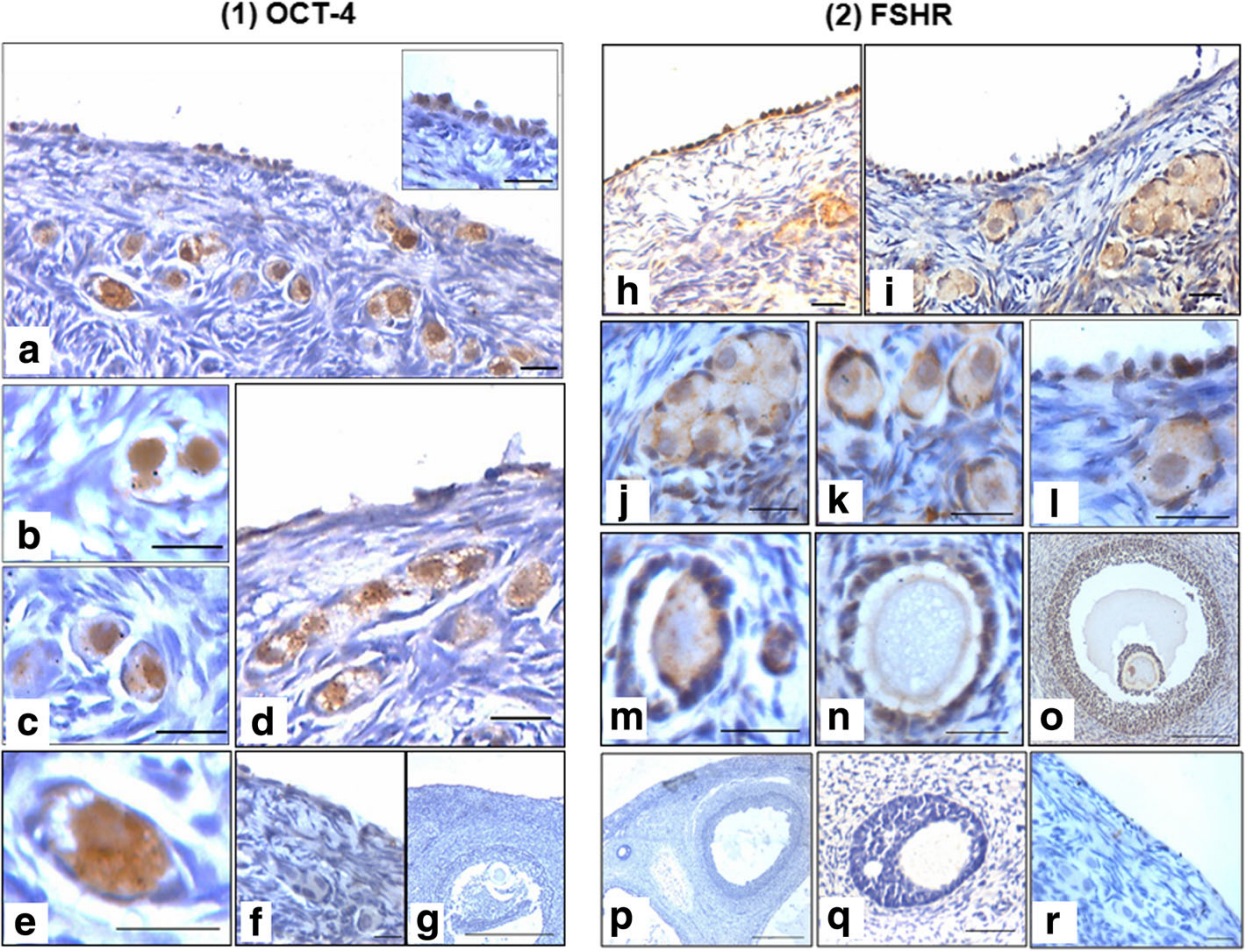
OCT-4 and FSHR immuno-localization on sheep ovarian sections. **a** Few cells on OSE were positive for OCT-4 (**b**-**d**) cluster of oocytes/oogonia in primordial and primary follicles and ooplasm were positive for OCT-4 whereas (**c**-**e**) surrounding granulosa cells and stromal cells were completely negative. Oocytes in large secondary and tertiary follicles in medulla region also showed OCT-4 expression whereas surrounding granulosa cells were negative. **f**-**g** Negative control with omission of primary antibody. **h**-**i** OSE cells show FSHR expression, (**j**) cluster of oocytes, (**k**-**m**) individual primordial and primary follicles oocytes nucleus and ooplasm both were positive for FSHR including surrounding granulosa cells, stromal cells were completely negative. **n**-**o** In medulla region larger oocytes & ooplasm including surrounding granulosa cells were positive for FSHR. **p** Negative control with omission of primary antibody (**q**-**r**) Negative control using peptide blocking of antibody

Figure 12 shows immuno-localization of OCT-4 (A-G) and FSHR (H-R) on ovarian sections. Positive staining for OCT-4 was observed in the OSE (A). Oogonial cluster and individual oogonia and oocytes in PF in ovarian cortex along with primary to secondary follicles and cohort of follicles also showed OCT-4 expression (B-D). Initially OCT-4 was observed in the nuclei of oogonia/ oocytes whereas in primary to secondary follicles, OCT-4 expression was in the ooplasm and the oocytes were negative (E). Granulosa cells in growing follicles did not express OCT-4. FSHR staining was observed in few cells in OSE along with oogonial cluster and individual oogonia/oocytes including surrounding granulosa cells positive for FSHR. In growing follicles, oocytes and granulosa cells expressed FSHR. Negative controls for this study including peptide blocking of FSHR antibody are provided (Additional file 1: Figure S9).

To conclude from this section, presence of oogonial cluster with incomplete cytokinesis in adult ovary is direct evidence in support of postnatal neo-oogenesis and primordial follicle assembly in ovarian cortex from the ovarian stem cells lodged in the OSE. PCNA expression in OSE cells was not expected if the adult ovary has fixed number of pre-formed primordial follicles at birth however, PCNA expression in stem/progenitor cells in adult ovarian sections in the present study is direct evidence in support of stem cells activity in the adult ovary that may be involved in neo-oogenesis and PF assembly. FSHR expression in growig follicles in the cortical tissue was also intriguing as it is currently understood that FSHR is expressed only on the granulosa cells of growing follicles whereas PF are gonadotropin independent [36]. OCT-4 and FSHR expression in stem cells/oocytes and oogonia in newly assembled follicles suggest that neo-oogenesis and primordial follicle assembly occurs in response to circulatory FSH.

## Discussion

Results of the present study provide additional evidence in support of the presence of two distinct populations of stem cells including pluripotent VSELs and progenitors OSCs localized in the OSE in adult sheep ovary in agreement with our earlier published data [5, 10–14]. VSELs and OSCs are spherical cells with high nucleo-cytoplasmic ratio and can be distinguished from each other based on their size and OCT-4 expression which is nuclear in VSELs and cytoplasmic in OSCs. Both VSELs and OSCs express FSHR and VSELs undergo ACD to self-renew give rise to OSCs that undergo SCD and clonal expansion to form germ cell nests in response to FSH treatment may be via alternately spliced FSHR3 isoform confirming our earlier data [11]. Expression of FSHR on stem cells (besides granulosa cells) was further confirmed in the present study by different approaches (immuno-phenotyping, IF, RT-PCR). Also pre-incubating FSHR antibody with specific peptide (against which it was raised) during immuno-localization studies completely abrogated FSHR expression thus proving that FSHR expression on the stem cells is specific. Co-expression of pluripotent stem cell markers SSEA-4/OCT-4 with FSHR on stem cells in OSE cells smears provide additional evidence to support the presence of pluripotent stem cells in OSE that express FSHR. Evidence is also provided for the first time in the literature that most primitive, pluripotent VSELs undergo asymmetric cell division (ACD) to give rise to the OSCs which in turn undergo symmetric cell division and clonal expansion to form a germ cell nest. VSELs and OSCs are of unequal size, have different fate and show distinct gene expression of OCT-4 and NUMB similar to our earlier results on bone marrow [21] and testis [17]. These results and effect of FSH on ovarian stem cells to give rise to germ cell nests-like structures are in contrast to the findings of Lei and Spradling [37] who failed to detect stem cells activity and germ cell nests in adult ovary. The reasons why they failed to detect stem cells and germ cell nests in adult ovary were discussed [38]. We have earlier reported similar spheres in chemoablated mouse ovary after FSH treatment [12]. Formation of these clusters with cytoplasmic connectivity or ‘nests’ is not characteristic of only ovarian stem cells but is the property of stem cell derived tissue-specific progenitors and germ cell nests represent sphere formation like mammosphere, cardiosphere, neurosphere etc. before initiating differentiation.

Present study also provides further direct evidence in support of postnatal neo-oogenesis and primordial follicle assembly in adult sheep ovary and that it possibly occurs in response to FSH. If the ovary is endowed with a fixed number of primordial follicles at birth, one would not expect to observe PCNA expression in oogonia/oocytes in the ovarian cortex of adult sheep ovary. On the contrary, we observed PCNA expression in oogonia/oocytes in primordial follicles in the ovarian cortex. In addition, a distinct staining for OCT-4 and FSHR along with PCNA was also observed on the stem cells lodged in the OSE which differentiate into oogonia/oocytes. OCT-4 positive stem cells in OSE may proliferate in response to FSH and undergo clonal expansion to form ‘spheres’ or ‘nests’, meiosis, GCN breakdown and PF assembly in adult sheep ovaries. We have earlier reported similar activity of stem cells in adult mouse OSE/ovarian cortex on treatment with PMSG [34].

Similar to various stages described in fetal ovary [39, 40], we have shown that adult ovarian cortex harbor germ cell nests comprising of a cluster of oogonia with continuous cytoplasm and surrounded by a single layer of granulosa cells. Germ cell nest breaks down and the surrounding somatic cells surround the oocytes to assemble as individual primordial follicles. That the cells in the nest arise from the stem cells was confirmed (i) by in vitro study wherein FSH treatment stimulated the stem cells to undergo asymmetric/symmetric cell divisions and clonal expansion and (ii) by expression of PCNA/OCT-4/FSHR on stem cells and the differentiated oogonia in ovarian sections.

Thus besides the currently held view that FSH acts on the granulosa cells in the ovary and on Sertoli cells in the testis, FSH receptors are also expressed on the stem cells (VSELs, OSCs/SSCs) in the gonads and FSH exerts a direct action resulting in their proliferation [11, 17]. It is widely accepted that FSH exerts its action on the granulosa cells of growing pre-antral follicle whereas initial follicle growth is gonadotropin independent. However, emerging direct action of FSH on stem cells (which also express FSHR) is indeed intriguing. Also the VSELs and HSCs in the hematopoietic system also express FSH and sex hormone receptors [25–28]. Presence of FSHR on granulosa cells alone in the ovary cannot explain FSHR expression on ovarian cancer cells whereas FSHR expression on the stem cells in the OSE can explain why more than 90% of ovarian cancers express FSHR and arise from the epithelial cells lining the ovary and fimbriae epithelium. Auresperg has earlier reported that stem cell profile of OSE is reproduced in the oviductal fimbriae [41]. Ovarian cancers possibly arise from stem cells which express FSHR and are located in the OSE [42] and this was recently confirmed by Virant-Klun’s group [43].

A careful review of literature reveals that primers selection for RT-PCR and in situ hybridization from different exons of FSHR gene and presence of alternatively spliced FSHR isoforms led to the erroneous conclusion that initial follicle growth is gonadotropin independent. Earlier studies in late nineties in sheep [44] and humans [45] used techniques like in situ hybridization and RT-PCR to study FSHR expression on ovarian follicles of different stages of development. FSHR mRNA was observed only in growing follicle in both sheep and humans and not in the early primordial follicles [44, 45]. In contrast, other groups reported FSHR on OSE [46, 47] and on oocytes [48, 49] and also on carcinoma cells [50–52]. Interestingly Zheng et al. [46] used primer sequences from exons 1–5 (which are common in both FSHR1 and FSHR3) of FSHR gene to demonstrate FSHR in the OSE. Meduri et al. [53] showed FSHR (not LHR) binding to human oocytes of primary follicles at both protein and mRNA level (20 folds more than on granulosa cells). FSH binding to oocytes was shown by autoradiography, oocytes responded by mobilization of Ca2+ and authors suggested a direct action of FSH on oocytes development. They used anti FSHR antibody raised against the extracellular domain of FSHR and a 366 bp FSHR transcript (using primers from exons 7 and 10) was detected in granulosa cells whereas a 191 bp product against FSHR (using primers from exons 2 and 4) was detected in the oocytes.

Thus it is very evident that alternatively spliced FSHR transcripts are involved in FSH action and both Oktay [45] and Tisdall [44] studies missed out on FSHR expression in primordial follicles since they used primers spanning exons 8–10 for RT-PCR and in situ hybridization which are specific to G-protein coupled, canonical FSHR1 receptor and are spliced out in alternatively spliced growth factor type 1 FSHR3 receptor [24]. This is the reason why these groups did not detect FSHR on PF in contrast to studies by Zheng [46] and Meduri [53] discussed above. FSH-R3 signaling pathway includes cAMP-independent activation of ERK downstream of an SNX-482 sensitive component likely to be the Cav2.3 calcium channel. Using sheep ovaries, Sullivan et al. [54] reported that Fshr3 is the predominant transcript in small, medium and pre-ovulatory follicles and not the canonical Fshr1. Similarly, Babu et al. [55] reported that PMSG treatment to 21 days old mouse induced higher up-regulation of Fshr3 compared to Fshr1 and a band of 39 kDa was detected using an antibody specific to Fshr3 by Western blotting (in contrast to 78 kDa band generally expected for canonical FSHR1). Our results demonstrate that in both testes [17] and ovaries [11], FSH exerts its action on the stem/germ cells via alternatively spliced Fshr3 receptor which gets more up-regulation compared to Fshr1. Using the same FSHR antibody raised against exon 2 which is conserved in alternately spliced isoforms, we recently detected all the 4 FSHR isoforms in mouse uterus [56].

Several groups [57–59] in the past have reported effect of FSH treatment on OSE. FSH was also demonstrated to be involved during PF formation in hamster ovary [60]. We have also reported that numbers of PF cohorts in the ovarian cortex are increased after PMSG treatment [34]. This was associated with an increase in pluripotent transcripts (Oct-4A, Nanog), meiosis (Scp-3) and germ cells (Oct-4B, Mvh) specific markers. MVH showed positive immuno-staining on germ cell nest-like clusters and at places primordial follicles appeared connected through oocytes/oogonia. The stem cells are activated in response to FSH treatment and also undergo meiosis. These results were supported by studies undertaken in vitro wherein human and marmoset cortical tissue was cultured in presence and absence of FSH [35]. Large numbers of ovarian stem cells were released on the cell culture insert and could spontaneously differentiate into oocytes-like structures [61]. qRT-PCR analysis revealed that treating cortical tissue pieces with FSH (0.5 IU/ml) or bFGF(100 ng/ml) for 3 days resulted in an increased expression of gene transcripts specific for VSELs (Oct-4A, Nanog), OSCs(Oct-4), early germ cell (c-Kit, Vasa) and PF growth initiation (oocytes-specific Gdf9, Lhx8 and granulosa cells specific Amh) suggestive of follicular transition [35]. Also Parte et al. [61] showed the presence of germ cell nests, Balbiani body-like structures and cytoplasmic streaming extensively described during fetal ovary development, are indeed well recapitulated during in vitro oogenesis in adult OSE cultures.

Several lines of evidence exist to support our results suggesting a unique role of FSH during PF formation. A closer examination of FSHR knockout ovary shows significant reduction in pool of primordial and primary follicle compared to normal mice ovary follicle count [62, 63]. Possibly certain compensatory mechanisms or altered cell signaling pathways may be functional and result in PF assembly. O’Shaughnessy et al. [64] have reported that FSHR mRNA and alternate splicing is detectable in day 1 ovaries (with only PF) and increase further on Day 5 (when primary follicles first appear). In hypogonadal (hpg) mice which lack circulating gonadotropins show normal levels of FSHR mRNA up to D15 suggesting the existence of compensatory mechanisms for FSH actin. Allan et al. [65] have reported that FSH treatment to mature hpg mice increases the primordial follicles reserve. Roy and Albee [60] reported that FSH is crucial for PF formation in hamster ovaries as well. Injecting FSH-specific polyclonal antibody in pregnant hamsters resulted in dramatic loss of PF and this effect was reversed by treatment with FSH analog (PMSG). Lei et al. [66] reported that in mouse ovaries (postnatal days 1–3),

FSH facilitates germ cell nest breakdown and PF formation. Results of present study are stimulating but at the same time still in a preliminary stage. It will be a challenge to demonstrate neo-oogenesis and follicular assembly in adult ovary in vivo and newer models need to be developed to detect the same. A recent study [67] has demonstrated that transplanted female germ line stem cells can restore function of POF ovary and generate offspring. Our results suggests that similar to testicular stem cells [15], ovarian VSELs also undergo asymmetric cell divisions (ACD) whereas OSCs undergo symmetric cell divisions (SCD) and clonal expansion to form germ cell nests. Expression of FSHR on stem cells and the possibility that FSH may exert a direct action on ovarian stem cells leading to neo-oogenesis and follicle assembly provides a paradigm shift in our current understanding of FSH action on ovaries.

## Conclusions

To conclude, adult ovary harbors two distinct populations of stem cells including VSELs and OSCs in the OSE and is as dynamic in nature as the testis producing gametes throughout life rather than harboring fixed pool of gonadotropin independent primordial follicles. We show for the first time, that VSELs are the most primitive pluripotent stem cells with nuclear OCT-4 that self-renew and give rise to slightly bigger OSCs with cytoplasmic OCT-4 by undergoing ACD. These OSCs in turn undergo SCD and clonal expansion to form GCN. GCN breakdown and undergo further differentiation into oocytes and PF assembly. The stem cells express FSHR and respond directly to FSH. The existing concept that initial PF growth is gonadotropin independent is possibly incorrect and this confusion occurred due to presence of FSHR isoforms which requires careful primer designing for RT-PCR studies. Also data is generated how the stem cells possibly act under normal physiological conditions to form PF. The study provides huge scope for further research on the involvement of stem cells during neo-oogenesis and PF assembly in adult ovary and also provides novel understanding leading to menopause and cancer and novel targets for fertility regulation.

## Additional file

Additional file 1: Figure S1. The presence of two stem cell population in OSE by flow cytometry analysis is shown. **Table S1.** List of antibodies used in the study. **Table S2.** List of primers used in the study. **Figure S2.** H & E staining of freshly isolated sheep OSE smears (arrows represent small putative VSELs smaller then RBCs and asterisk represent OSCs and cell doublets (circled) show dividing stem cells). **Figure S3.** RT-PCR on freshly isolated OSE cells and granulosa cells surrounding oocytes. **Figure S4.** FSHR expression on ovarian stem cells. **Figure S5a.** Z stack of OCT-4 expressing germ cell clusters in FSH treated OSE cell culture. **Figure S5b.** Z stack of FSHR expressing germ cell clusters in FSH treated OSE cell culture. **Figure S6.** Co-expression of FSHR and SSEA-4 on ovarian stem cells. **Figure S7.** FSHR expression on proliferating and dividing ovarian stem cells cultured in vitro within OSE cells on FSH treatment. **Figure S8.** H&E staining of ovarian sections (A) shows a distinct layer of OSE cells (B-C) PCNA immuno-localization on sheep ovarian sections showed few cells in OSE positive for PCNA, cluster of oocytes/germ cell cyst, individual primordial and primary follicles located in cortical region of ovary showed strong nuclear PCNA with faint stain in ooplasm. **Figure S9a.** Negative control for ICC to show specificity of staining (i) Negative for VASA (ii) Negative for SSEA-4 and PCNA (iii) Negative for FSHR and OCT-4. **Figure S9b.** Negative control by peptide blocking of FSHR antibody to show specificity of staining obtained using FSHR. (PDF 1911 kb)

## Abbreviations

ACD: asymmetric cell division
FSH: follicle stimulating hormone
FSHR: FSH receptors
GCN: germ cell nest
HSCs: hematopoietic stem cells
OSCs: ovary stem cells
OSE: ovary surface epithelium
PF: primordial follicles
SCD: symmetric cell division
SSCs: spermatogonial stem cells
VSELs: very small embryonic-like stem cells
