# Correction: Further characterization of adult sheep ovarian stem cells and their involvement in neo-oogenesis and follicle assembly

**DOI:** 10.1186/s13048-023-01139-9

**Published:** 2023-03-24

**Authors:** Hiren Patel, Deepa Bhartiya, Seema Parte

**Affiliations:** Stem Cell Biology Department, ICMR-National Institute for Research in Reproductive Health, Jehangir Merwanji Street, Parel, Mumbai 400 012 India

**Correction: J Ovarian Res 11, 3 (2018)**

10.1186/s13048-017-0377-5

The original article [1] contains errors in Figs. 3, 7 and Additional file Figure S5a. 2 sets of images in Fig. 3A were incorrectly a repeat of Fig. 4 in an earlier article by the authors [2]. In addition, Fig. S5a appeared to overlap with Fig. 2b in a previous article [3].

- Figure 3A has been corrected.
- Figure 7d has been corrected.
- Figure S5a legend was revised to include the reference to a previous paper as shown below.

**Figure S5a**. Z stack of OCT-4 expressing germ cell clusters in FSH treated OSE cell culture [3].

The correct Figs. 3 and 7 are shown below.

**Fig. 3:**
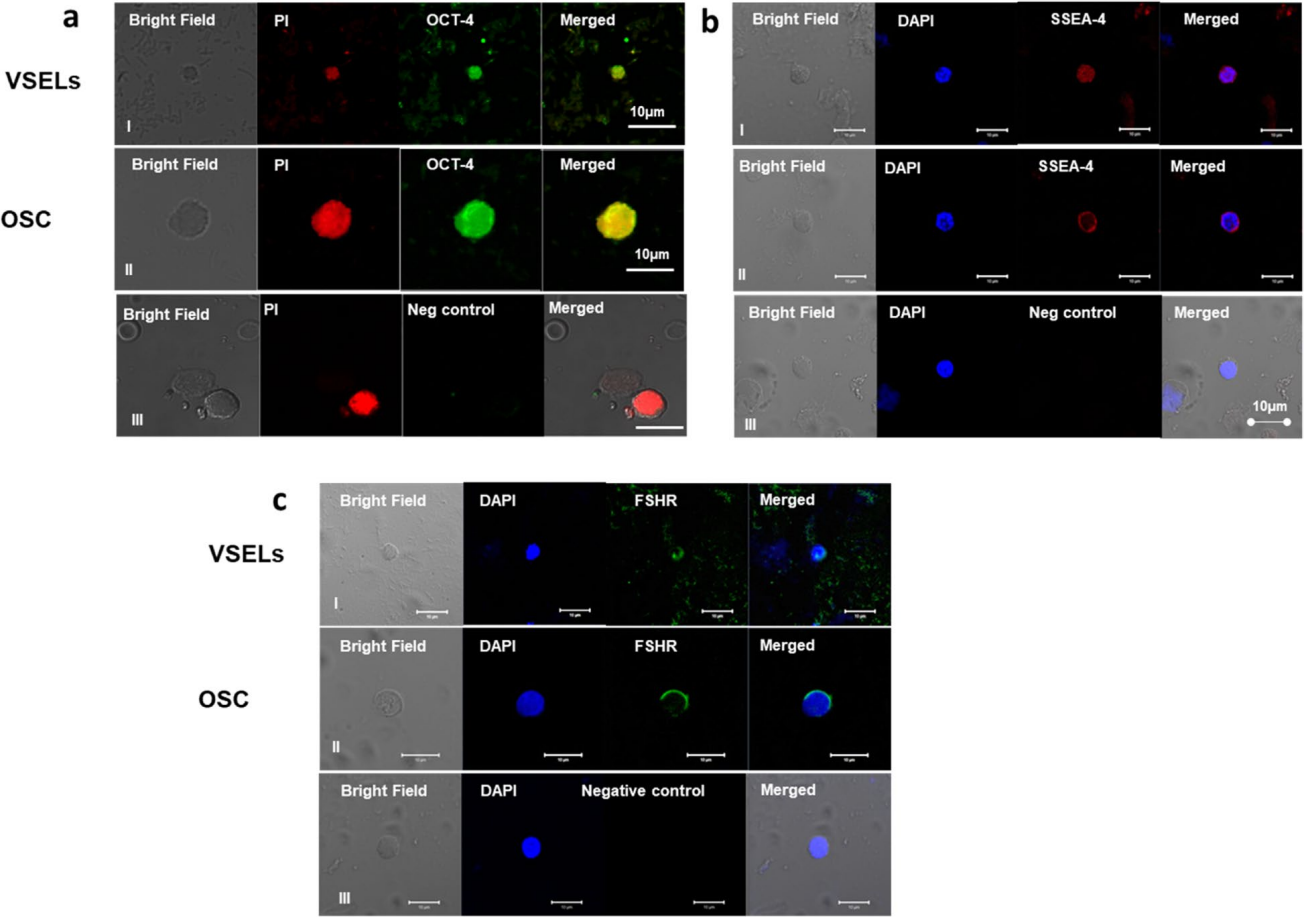
OCT-4, SSEA-4 and FSHR expression on ovarian stem cells. Cells with nuclear OCT-4 (Ai) and surface SSEA-4 (Bi) and FSHR (Ci) represent pluripotent VSELs, and slightly bigger cells with cytoplasmic OCT-4 (Aii) minimal surface SSEA-4(Bii) and FSHR (Cii) represent progenitor OSCs. Negative control by omission of primary antibody showed no staining (**a, b** & **c** iii)

**Fig. 7:**
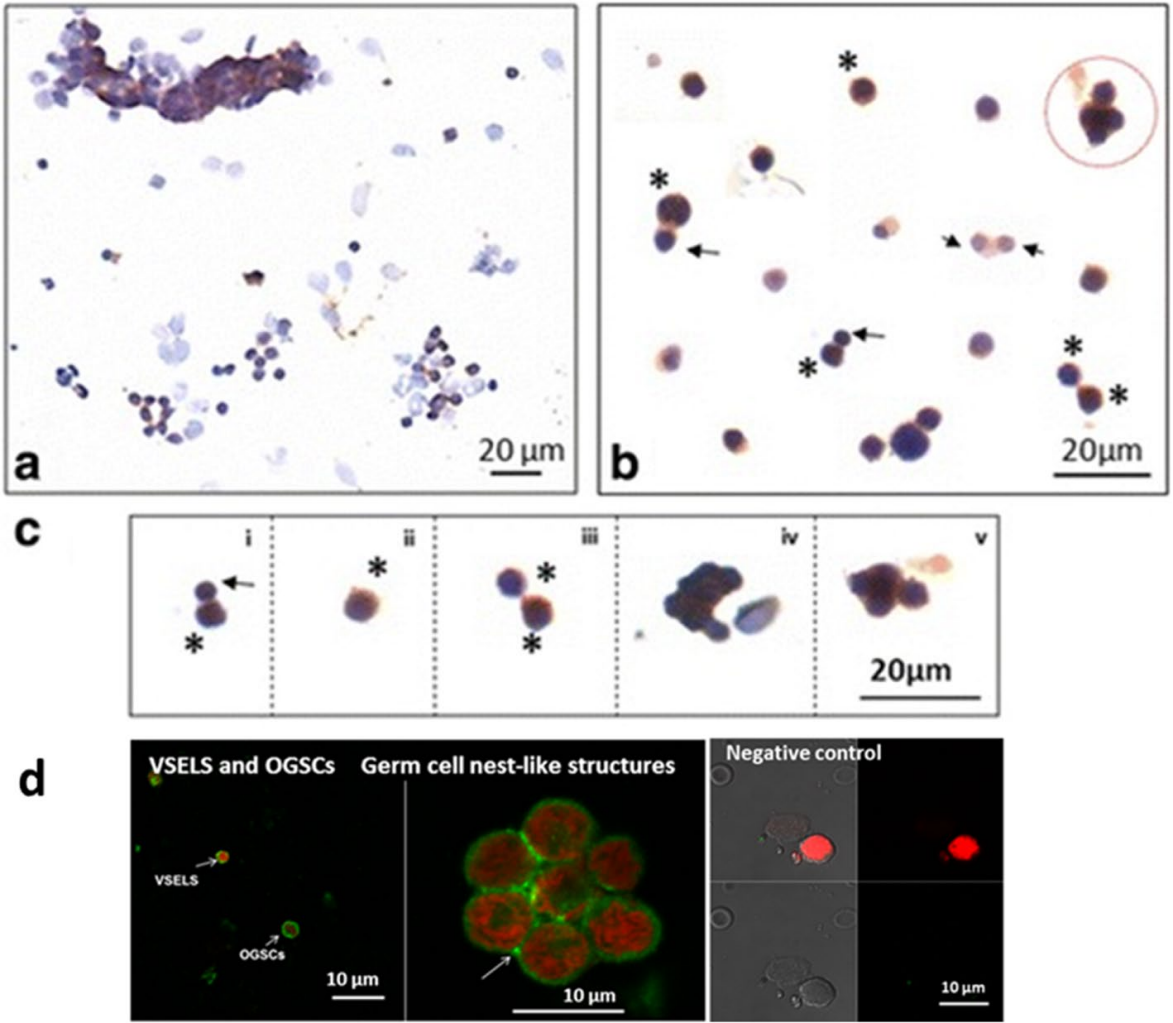
FSHR expression on cells obtained by scraping sheep ovary surface after FSH treatment in vitro. **a** Low magnification showing epithelial cells and stem cells in close vicinity with FSHR expression only on the stem cells. **b** Various fields were photographed to study stem cells division. Two distinct size of FSHR positive stem cells were visualized including slightly small VSELs (arrow) and bigger OSCs (asterix). Both asymmetric and symmetric cell divisions and germ cell nest-like structures (circled) were clearly visualized. Please note that both (**a**) and (**b**) are actually composites prepared by putting together various fields as these cells are spread far apart on the slides. **c** Stem cells are linearly arranged to understand their biology. (i) Small sized VSEL undergoes asymmetric cell division to give rise to slightly bigger OSCs which (ii) OSC (iii) undergo symmetric cell division (iv-v) and clonal expansion with incomplete cytokinesis to form a germ cell nest-like structure. Similar germ cell nest-like structures in adult ovary have been reported earlier also [6, 7]. **d** Representative confocal images showing FSHR expression on VSELs/OSCs/germ cell nest-like structure and negative control

The authors sincerely apologize for the errors. The errors do not affect the conclusion of the article.
