# Supplementary material for: Further characterization of adult sheep ovarian stem cells and their involvement in neo-oogenesis and follicle assembly
Source: J Ovarian Res. 2018 Jan 5;11:3. doi: 10.1186/s13048-017-0377-5 (PMC5755409; doi:10.1186/s13048-017-0377-5)
Supplement: Additional file 1: Figure S1. — The presence of two stem cell population in OSE by flow cytometry analysis is shown. Table S1. List of antibodies used in the study. Table S2. List of primers used in the study. Figure S2. H & E staining of freshly isolated sheep OSE smears (arrows represent small putative VSELs smaller then RBCs and asterisk represent OSCs and cell doublets (circled) show dividing stem cells). Figure S3. RT-PCR on freshly isolated OSE cells and granulosa cells surrounding oocytes. Figure S4. FSHR expression on ovarian stem cells. Figure S5a. Z stack of OCT-4 expressing germ cell clusters in FSH treated OSE cell culture. Figure S5b. Z stack of FSHR expressing germ cell clusters in FSH treated OSE cell culture. Figure S6. Co-expression of FSHR and SSEA-4 on ovarian stem cells. Figure S7. FSHR expression on proliferating and dividing ovarian stem cells cultured in vitro within OSE cells on FSH treatment. Figure S8. H&E staining of ovarian sections (A) shows a distinct layer of OSE cells (B-C) PCNA immuno-localization on sheep ovarian sections showed few cells in OSE positive for PCNA, cluster of oocytes/germ cell cyst, individual primordial and primary follicles located in cortical region of ovary showed strong nuclear PCNA with faint stain in ooplasm. Figure S9a. Negative control for ICC to show specificity of staining (i) Negative for VASA (ii) Negative for SSEA-4 and PCNA (iii) Negative for FSHR and OCT-4. Figure S9b. Negative control by peptide blocking of FSHR antibody to show specificity of staining obtained using FSHR. (PDF 1911 kb) [file 13048_2017_377_MOESM1_ESM.pdf]

Additional file

Further Characterization of Adult Sheep Ovarian Stem Cells and Their Involvement during Neo-oogenesis and Follicle Assembly

Hiren Patel, Deepa Bhartiya and Seema Parte, Stem Cell Biology Department, ICMR-National Institute for Research in Reproductive Health, Jehangir Merwanji Street, Parel, Mumbai, INDIA

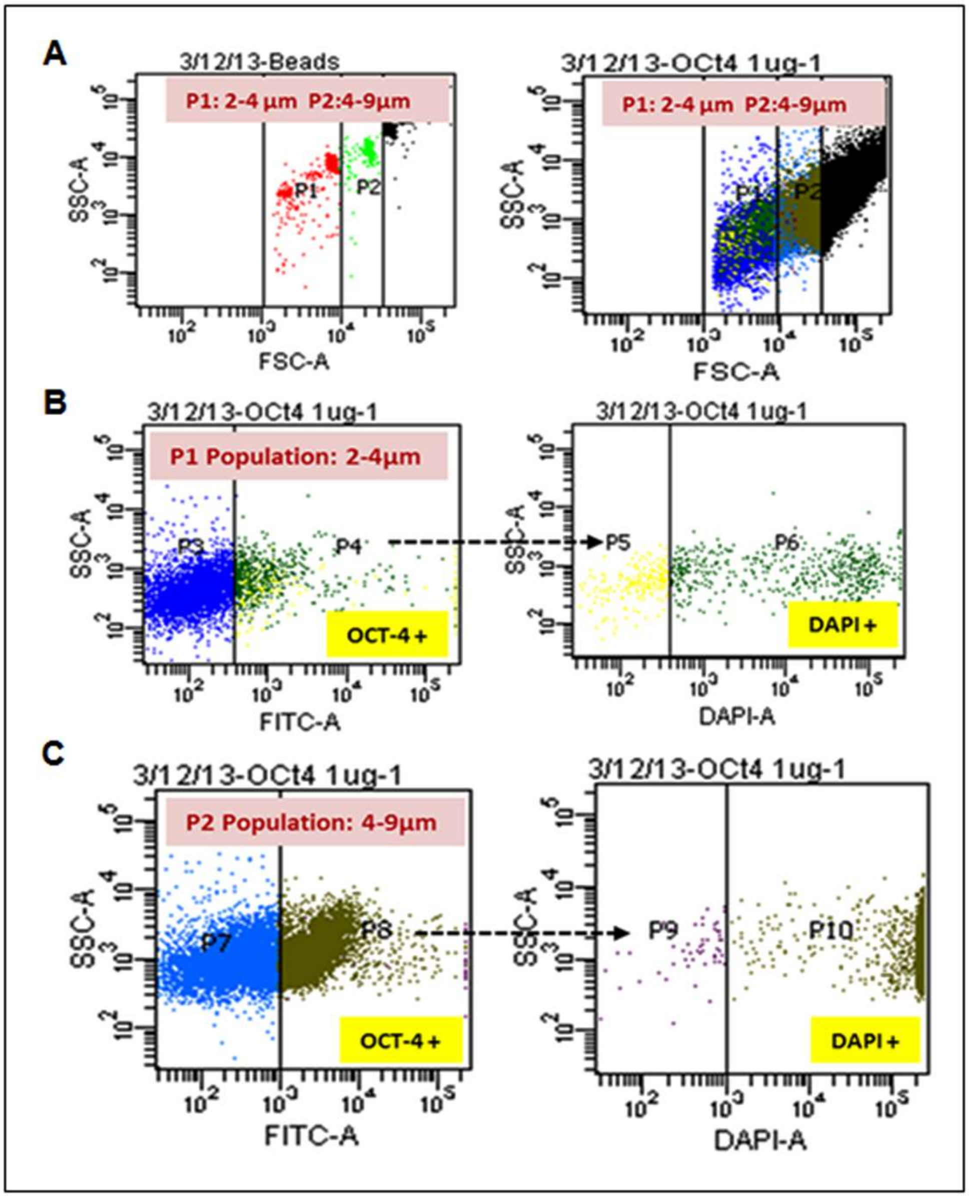

Figure S1: The presence of two stem cell population in OSE by flow cytometry analysis is shown. (Upper panel) The OSE cells are segregated in two populations (Figure 1A) in the range of 2-4 µm and 4- 9 µmusing size calibrated beads as reference to study OCT-4 positive cells. (Middle panel) Interestingly on analysis we found 1.2% of OCT-4 positive cell in size range 2-4 µm that were DAPI positive indicating presence of small putative stem cells VSELs in OSE and not debris. (Bottom panel) 6.86% of bigger stem cells population in size range of 4-9 µm was also positive for OCT-4 and DAPI. Overall analysis of total population in size range of 2-9 µm showed average 4% of ovarian stem cell in OSE positive for OCT-4 and DAPI, similar to published data. [Reproduced from ref 14].

Table S1: List of antibodies used in the study

Sr.no	Antibody	Description
1	OCT-4, Abcam, Cambridge, UK ab19857	▪ anti-human OCT-4 antibody<br>▪ raised in Rabbit (polyclonal)<br>▪ reacts with mouse, sheep, human, rhesus monkey<br>▪ Isotype IgG<br>▪ IF and IHC dilutions (1:100µl)<br>▪ Flow cytometer (1µg for 1X 106 cells/ mL)<br>▪ Secondary for IF: goat anti-rabbit IgG Alexa flour 488 (Molecular Probes, Invitrogen, USA) Dilution: (1:1000 µl).<br>▪ Secondary ICC/IHC: Vectastain Elite ABC kit, Rabbit IgG Vector Laboratories Inc, California, USA) used as mentioned in data sheet.
2	OCT-4 PerCP-Cy tagged, BD Pharmingen™ USA (562251)	▪ anti-human OCT-4A Isoform<br>▪ Raised in mouse<br>▪ Isotype IgG_1<br>▪ Flow cytometer (1µg for 1X 106 cells/ mL).
3	FSHR Abcam, Cambridge, UK ab150557	▪ anti-human FSHR antibody<br>▪ raised in Rabbit (polyclonal)<br>▪ reacts with mouse, sheep, human, rhesus monkey<br>▪ Isotype IgG<br>▪ IF and IHC dilutions (1:100µl)<br>▪ Flow cytometer (1µg for 1X 106 cells/ mL)<br>▪ Secondary: goat anti-rabbit IgG Alexa flour 488 (Molecular Probes, Invitrogen, USA) Dilution: (1:1000 µl).<br>▪ Secondary ICC/IHC:Vectastain Elite ABC kit, Rabbit IgG Vector Laboratories Inc, California, USA) used as mentioned in data sheet.
4	PCNA, Sigma, USA P8825	▪ monoclonal antibody<br>▪ raised in mouse<br>▪ Isotype IgG_{2a}<br>▪ IHC dilutions (1:3000µL)<br>▪ Secondary: Vectastain Elite ABC kit, Mouse IgG Vector Laboratories Inc, California, USA). used as mentioned in data sheet.
5	SSEA-4, BioLegend CA, USA (330402) MC-813-70.	▪ anti-human SSEA-4 antibody<br>▪ Produced in mice<br>▪ Isotype: Mouse IgG3, κ<br>▪ Cross-Reacts: Mouse<br>▪ IF dilutions: (1:100µl)<br>▪ Secondary: goat anti-mice IgG Alexa flour 568 (Molecular Probes, Invitrogen, USA) Dilution: (1:1000 µl).<br>▪ Secondary: Vectastain Elite ABC kit, Mouse IgG Vector Laboratories Inc, California, USA) used as mentioned in data sheet.
6	VASA R&D Systems, USA, AF2030	▪ anti-human, polyclonal antibody<br>▪ raised in goat<br>▪ Isotype: IgG<br>▪ IF and ICC dilutions: (1:100µl)

		▪ Secondary: Vectastain Elite ABC kit, Goat IgG Vector Laboratories Inc, California, USA). used as mentioned in data sheet.
7	NUMB Abcam, Cambridge, UK ab14140	▪ Anti-human, polyclonal antibody<br>▪ raised in rabbit<br>▪ Isotype: IgG<br>▪ Cross-Reacts: Mouse, Rat, Human, Zebrafish, Monkey etc<br>▪ IF dilutions: (1:200µl)<br>▪ Secondary for IF: goat anti-rabbit IgG Alexa flour 488 (Molecular Probes, Invitrogen, USA) Dilution: (1:1000 µl).
8	OCT-4A [POU5F1], clone 7F9.2, Merck Millipore, Germany Catalogue No. MAB4419	▪ Anti-human, monoclonal antibody<br>▪ Raised in mice<br>▪ Isotype: IgG1κ<br>▪ Cross-Reacts: Human, Mouse<br>▪ IF dilutions: (1:100µl)<br>▪ Secondary for IF: goat anti-mice IgG Alexa flour 568 (Molecular Probes, Invitrogen, USA) Dilution: (1:1000µl).

OCT-4 antibody

Octamer-4 (OCT-4) is a member of the POU family of transcription factors and vital for the self-renewal of pluripotent stem cells. OCT-4 can generate three isoforms by alternative splicing, termed OCT4A, OCT4B and OCT4B1. OCT-4A has been confirmed as nuclear transcription factor responsible for pluripotent state whereas cytoplasmic OCT4B and OCT-4 B1 has no biological function has been associated with stemness and stress but its function remains unclear.

- <u>OCT-4, ab19857 antibody from Abcam:</u> It is anti-human polyclonal antibody raised in rabbit and able to detect both the alternatively spliced OCT-4 isoforms including OCT-4A in the nucleus of pluripotent stem cells and OCT-4B in cytoplasmic of progenitor stem cells. It detects both nuclear and cytoplasmic OCT-4.
- <u>OCT-4A MAB4419 antibody from Millipore:</u> It is anti-human monoclonal antibody raised in mice and detects only OCT-4A in the nucleus of pluripotent stem cells.
- <u>OCT-4 PerCP-Cy tagged antibody from BD Pharmingen (Cat no: 562251):</u> It is monoclonal antibody raised in mice and detects only OCT-4A in the nucleus of pluripotent stem cells. This OCT-4 antibody was used in flow cytometer and immunofluorescence technique to identify pluripotent VSELs as they express nuclear OCT-4A.

NUMB antibody (ab14140) from ABCAM

- Cells undergoing asymmetric cell division (ACD) express NUMB protein. During ACD, NUMB is asymmetrically distributed between the resulting daughter cells. The primitive stem cell express minimal or no expression of NUMB but committed progenitor uniformly expressed NUMB protein.
- In present study, NUMB was used to demonstrate self-renewal of pluripotent VSEL via ACD that give rise to OSCs in sheep OSE. NUMB was distinctly and uniformly expressed by progenitor OSC. In a dividing cell, NUMB is uniformly distributed in progenitor OSCs undergoing SCD but is gets asymmetrically segregated in one of the two cells which

undergo ACD and becomes specified to differentiate as it is implicated to suppress Notch signaling essential for maintaining undifferentiated stem cells. VSELs shows minimal or no expression of NUMB but committed progenitor OSCs uniformly expressed NUMB.

FSHR Antibody (ab150557) from Abcam:

- FSH receptor transcript undergoes alternative splicing to give rise to different isoforms by exon skipping. Four different alternatively spliced isoforms of FSHR are identified and reported. FSHR-1 (G protein-coupled form), FSHR-2 (dominant negative form), FSHR-3 (growth factor type-1), and FSHR-4 (soluble FSHR). Out of them, only two FSHR isoform (FSHR1 and FSHR3) has reported to have biological function. They have common N-terminus but variation in c-terminus, which provide different function. Canonical FSHR1 is a 75kDa member of the G-protein coupled receptor superfamily and has been found to be present on granulosa cells of the ovary in females and Sertoli cell of testis in males and responsible for steroidogenesis via the cAMP signal transduction pathway indirectly regulating growth, development and differentiation of germ cells. Whereas FSHR3 is a ~39kDa protein and has topology of a growth factor receptor and promotes DNA synthesis leading to proliferation via mitogen-activated protein kinase (MAPK) pathway, specifically the extracellular-regulated kinase (ERK) signaling cascade and voltage-dependent calcium channels and brings about proliferation of cells.

- In the present study, polyclonal commercial antibodies from extracellular domain (common to both FSHR1 and FSHR3 isoforms) were used (from Abcam, ab150557) for immune-localization studies. Co-localization of stem cell markers (SSEA-4 and OCT-4) with FSHR was studied by confocal microscopy to show that stem cells express FSHR. Specificity of FSHR expression was confirmed by peptide blocking experiment.

Table S2: List of primers used in the study

Sr. no	Genes	Sequences	Product size (bp)	Temp
1	Oct-4A	*F*: CAATTTGCCAAGCTCCTAAA<br>*R*: TTGCCTCTCACTTGGTTCTC	290 bp	53ºC
2	Oct-4	*F*: GAGCCGAACCCTGAGGAGTCCC<br>*R*: CAGCAGGGGCCGCAGCTTAC	225 bp	66ºC
3	Sox-2	*F*: TGATACGGTAGGAGCTTTGC<br>*R*: CTTTTGCCCCTTTAGAGACC	362 bp	56ºC
4	Stat3	*F*: TGGACAACATCATTGACCTG<br>*R*: CTGCTGCTTGGTGTAAGGTT	239 bp	55ºC
5	Vasa	*F*: AGGATGTTCCTGCATGGTTA<br>*R:* CTTGCAGGTTGTTTTCGTTT	210 bp	53ºC
6	Fshr1	*F*: CATTCACTGCCCACAACTTTCATC<br>*R*:TGAGTGTGTAATTGGAACCATTGGT	84 bp	60ºC
7	Fshr3	*F*: TCTCCACTGCTGCACTGTTGGGCT<br>*R*:ATTCAAATACAGGAAATAGAGAAA	382 bp	55ºC
9	Pcna	F: AGGACAGTGCCTTCATTTGGAC<br>R: TTGGCAAACGAATCACCCCA	150 bp	60ºC
10	Amh	F: CCTCAGTCGGACCGCAA<br>R:TTGCCTGTGTAGGCTGT	119 bp	55ºC
11	Gata-4	F:CTCGGAAGGCAGAGAGTGTG<br>R:GCCGGTTCTGTCCGTTCATC	125 bp	60ºC
12	Gapdh	F: GCCCAGAACATCATCCCTG<br>R: GGTCCTCAGTGTAGCCTAG	232 bp	60ºC

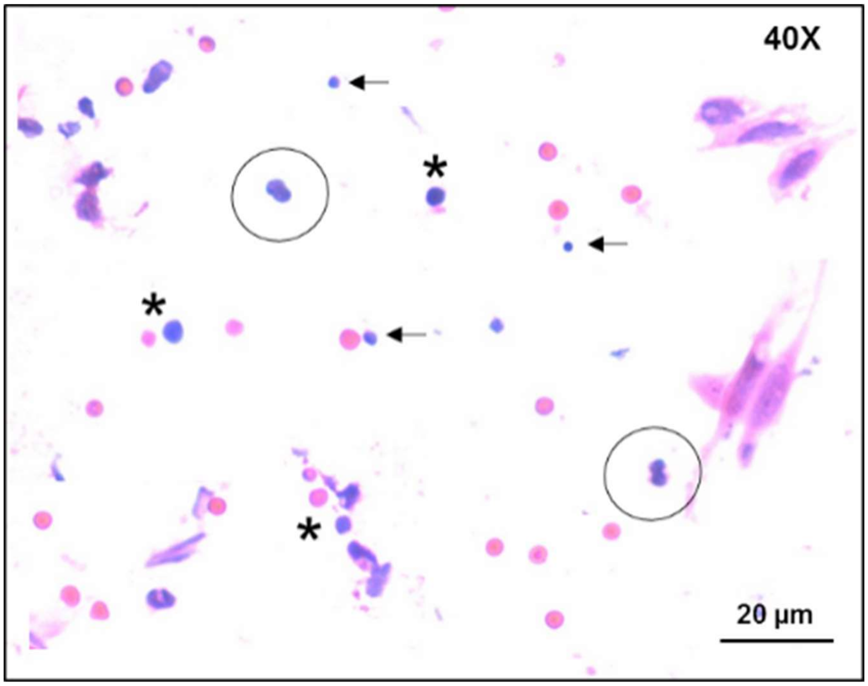

Figure S2: H & E staining of freshly isolated sheep OSE smears (arrows represent small putative VSELs smaller then RBCs and asterisk represent OSCs and cell doublets (circled) show dividing stem cells) . Pink colored RBCs devoid of nuclei are also clearly observed.

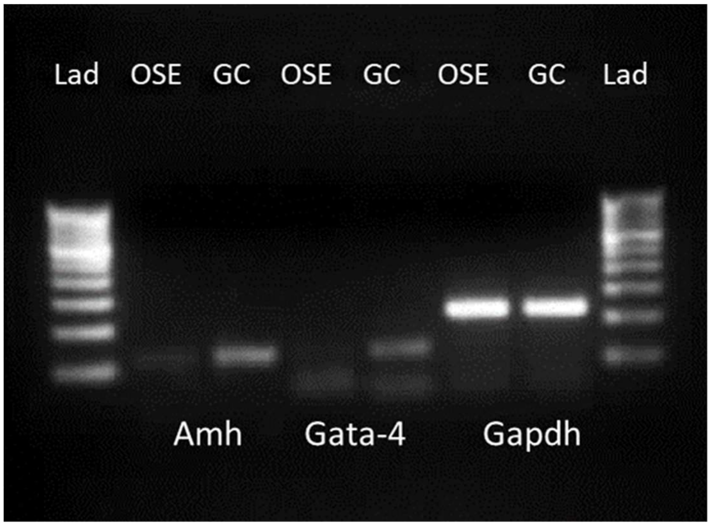

Figure S3: RT-PCR on freshly isolated OSE cells and granulosa cells surrounding oocytes. Results show no expression of granulosa cells markers Amh and Gata-4 in OSE, suggesting no contamination of granulosa cells in isolated OSE. Granulosa cells used as positive control showed amplification of both Amh, Gata-4 and Gapdh. Lad (ladder), OSE (freshly isolated ovarian surface epithelium), GC (granulosa cells), Amh (anti mullerian hormone)

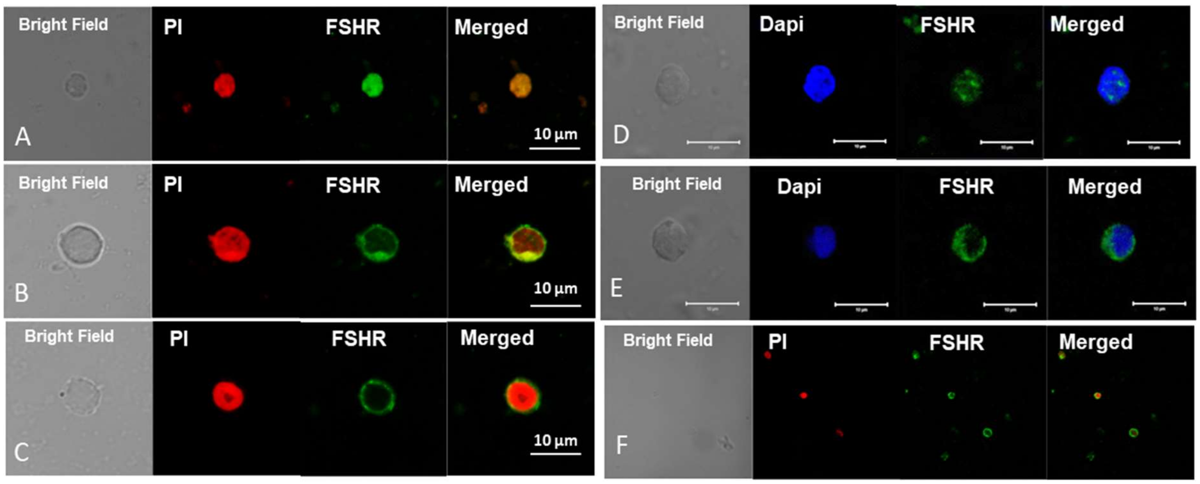

Figure S4: FSHR expression on ovarian stem cells.

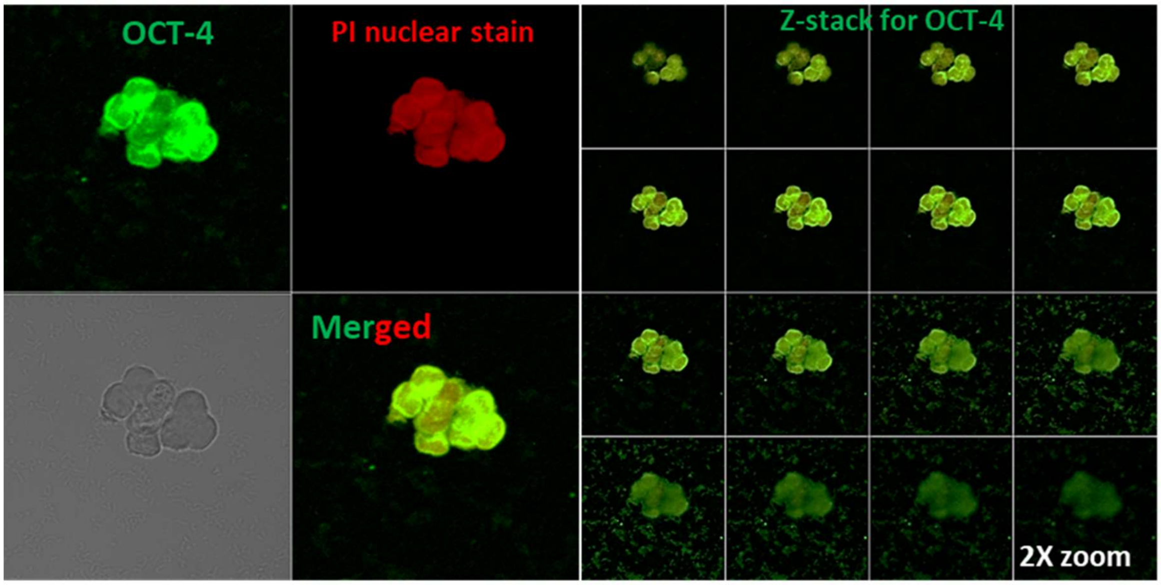

Figure S5a: Z stack of OCT-4 expressing germ cell clusters in FSH treated OSE cell culture.

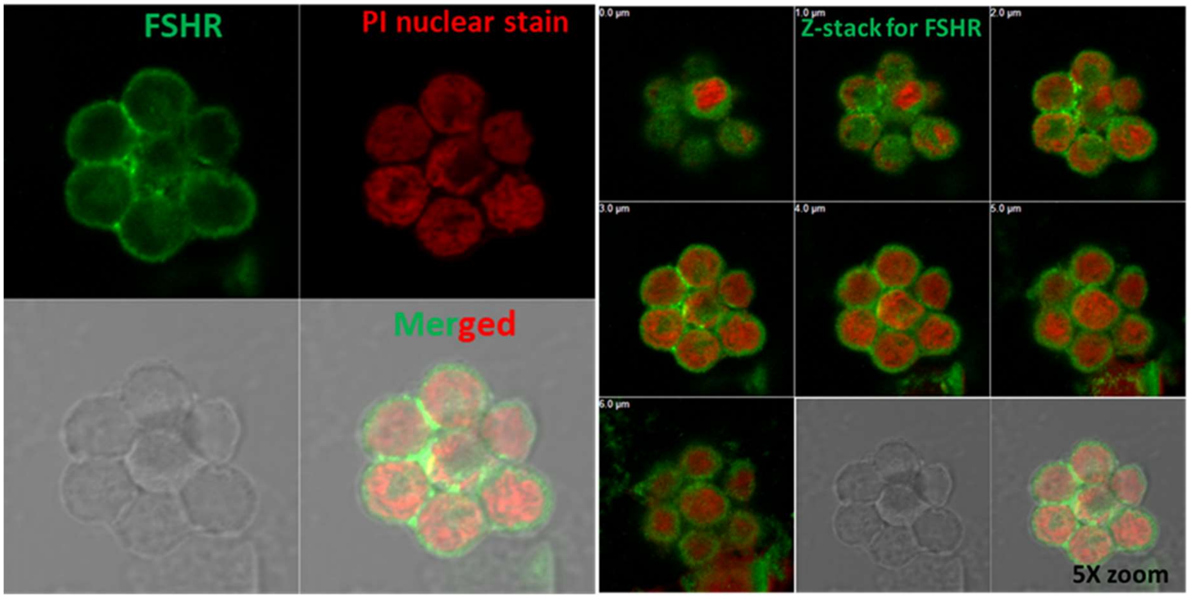

Figure S5b: Z stack of FSHR expressing germ cell clusters in FSH treated OSE cell culture.

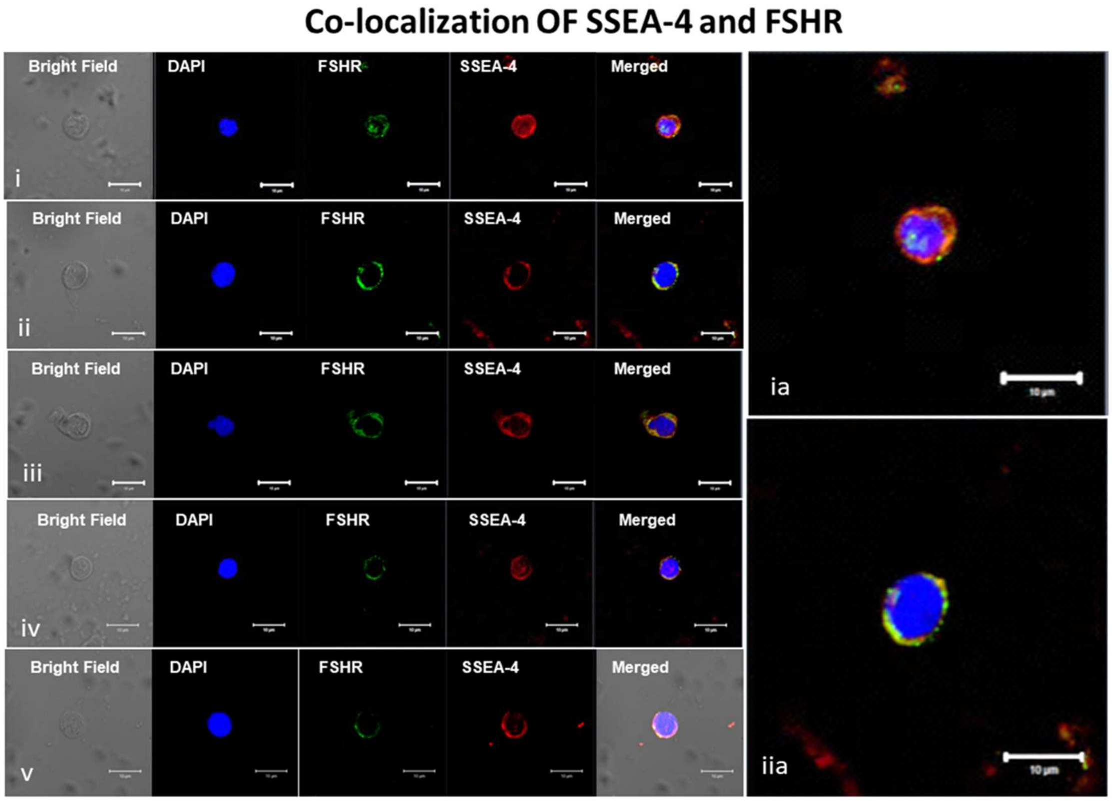

Figure S6: Co-expression of FSHR and SSEA-4 on ovarian stem cells

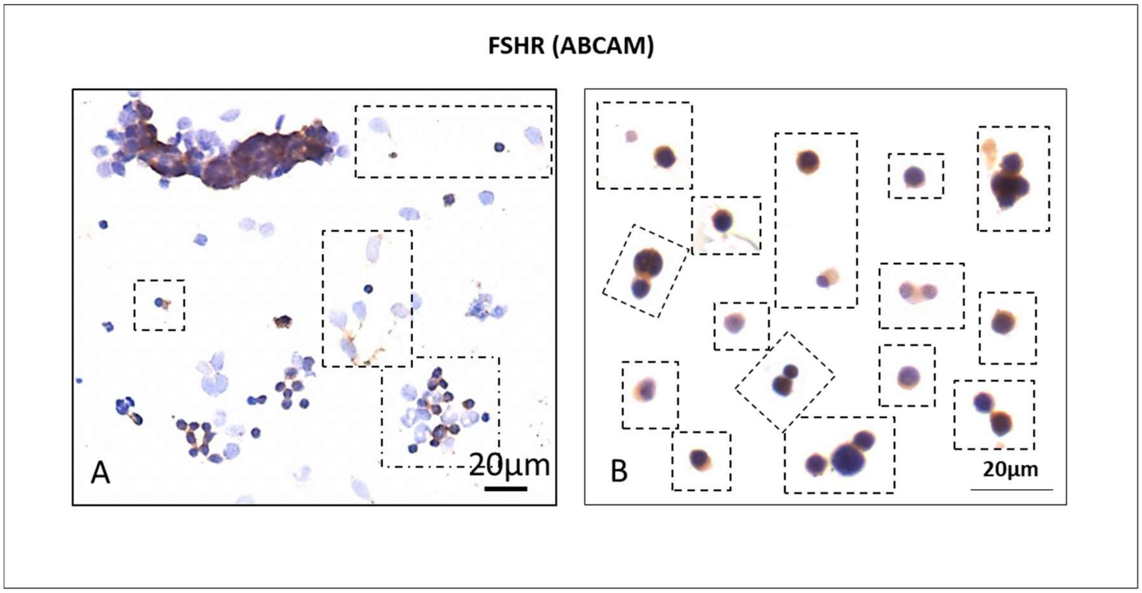

Figure S7: FSHR expression on proliferating and dividing ovarian stem cells cultured in vitro within OSE cells on FSH treatment.

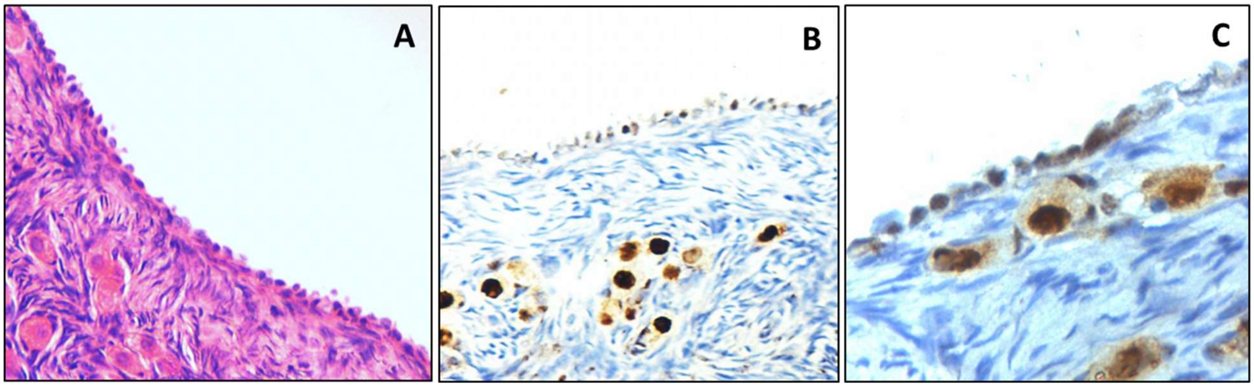

Figure S8: H&E staining of ovarian sections (A) shows a distinct layer of OSE cells (B-C) PCNA immuno-localization on sheep ovarian sections showed few cells in OSE positive for PCNA, cluster of oocytes/germ cell cyst, individual primordial and primary follicles located in cortical region of ovary showed strong nuclear PCNA with faint stain in ooplasm. Interestingly surrounding granulosa cells and stromal cells were negative for PCNA.

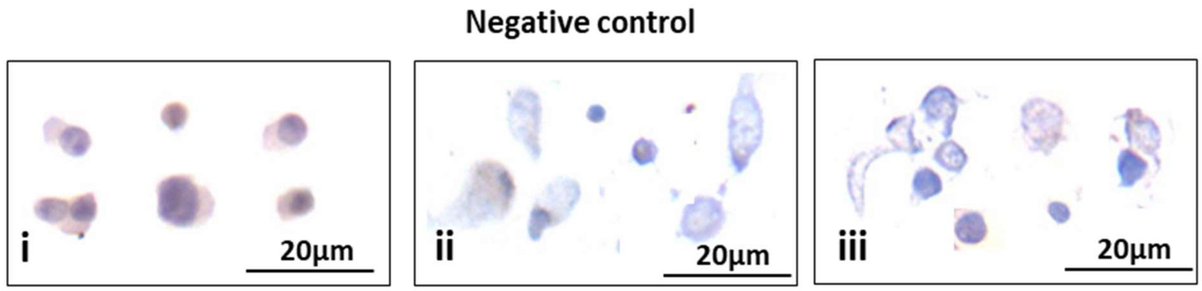

Figure S9a: Negative control for ICC to show specificity of staining (i) Negative for VASA (ii) Negative for SSEA-4 and PCNA (iii) Negative for FSHR and OCT-4.

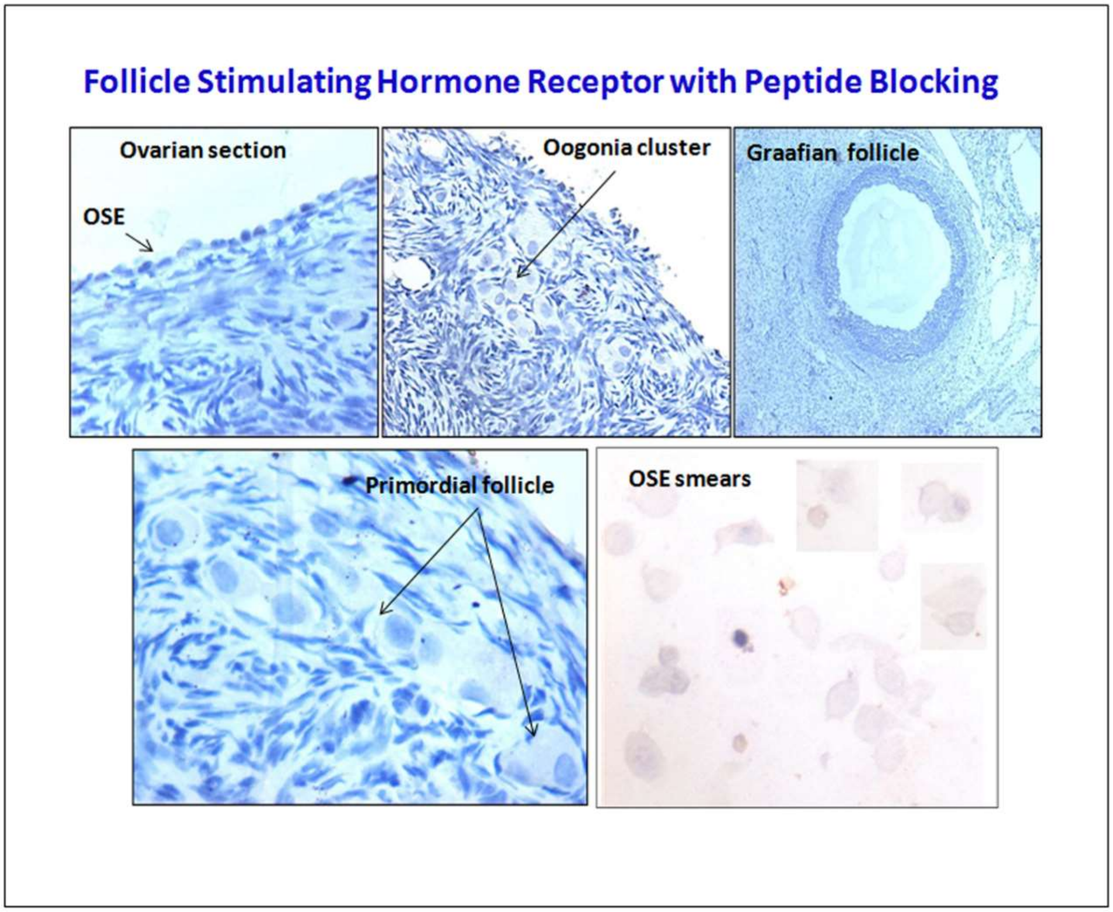

Figure S9b: Negative control by peptide blocking of FSHR antibody to show specificity of staining obtained using FSHR.
